# Alphaherpesvirus manipulates retinoic acid metabolism for optimal replication

**DOI:** 10.1016/j.isci.2024.110144

**Published:** 2024-06-04

**Authors:** Shengli Ming, Shijun Zhang, Jiayou Xing, Guoyu Yang, Lei Zeng, Jiang Wang, Beibei Chu

**Affiliations:** 1 College of Veterinary Medicine, Henan Agricultural University, Zhengzhou 450046, Henan Province, China; 2 Key Laboratory of Animal Biochemistry and Nutrition, Ministry of Agriculture and Rural Affairs, Zhengzhou, Henan Province 450046, China; 3 Key Laboratory of Animal Growth and Development of Henan Province, Henan Agricultural University, Zhengzhou 450046, Henan Province, China; 4 International Joint Research Center of National Animal Immunology, Henan Agricultural University, Zhengzhou 450046, Henan Province, China; 5 Ministry of Education Key Laboratory for Animal Pathogens and Biosafety, Zhengzhou 450046, Henan Province, China

**Keywords:** Virology, Cell biology

## Abstract

Retinoic acid (RA), derived from retinol (ROL), is integral to cell growth, differentiation, and organogenesis. It is known that RA can inhibit herpes simplex virus (HSV) replication, but the interactions between alphaherpesviruses and RA metabolism are unclear. Our present study revealed that alphaherpesvirus (HSV-1 and Pseudorabies virus, PRV) infections suppressed RA synthesis from ROL by activating P53, which increased retinol reductase 3 (DHRS3) expression—an enzyme that converts retinaldehyde back to ROL. This process depended on the virus-triggered DNA damage response, the degradation of class I histone deacetylases, and the subsequent hyperacetylation of histones H3 and H4. Counteracting DHRS3 or P53 enabled higher RA synthesis and reduced viral growth. RA enhanced antiviral defenses by promoting ABCA1- and ABCG1-mediated lipid efflux. Treatment with the retinoic acid receptor (RAR) agonist palovarotene protected mice from HSV-1 infection, thus providing a potential therapeutic strategy against viral infections.

## Highlights

• Retinoic acid (RA) synthesis is inhibited by viral infection
• Inhibition of RA synthesis by viral infection depends on DHRS3-DNA damage-P53 pathway
• RA promotes lipid efflux and antiviral innate immunity

## Graphical abstract

**Figure:**
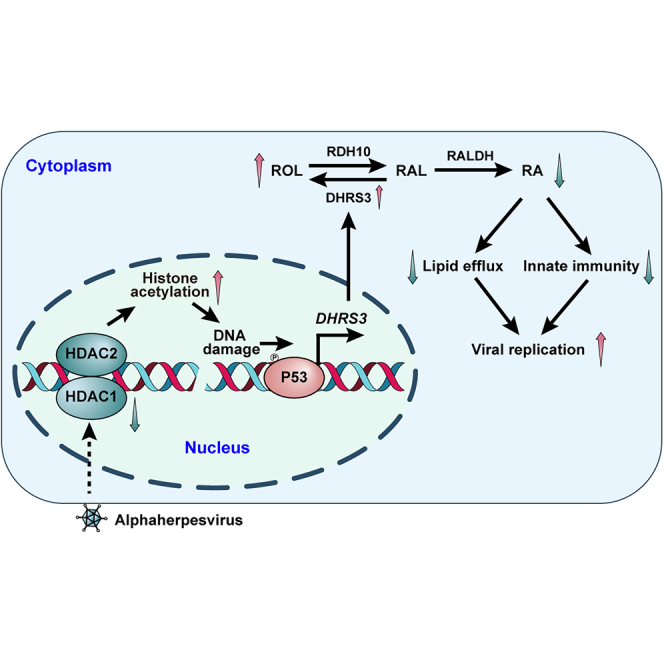

## Introduction

Although viruses are known to manipulate cell metabolism to facilitate optimal viral replication, the mechanisms involved in this process remain to be investigated.^1^^,^^2^ Substantial evidence has indicated that viruses commonly use glycolysis instead of oxidative phosphorylation to generate reducing equivalents and precursors for macromolecule biosynthesis.^3^^,^^4^ Increased nucleotides are another metabolic hallmark of viral infection.^5^ Viruses enhance their replication by stimulating lipid consumption and anabolism.^6^^,^^7^ However, how viruses modulate other metabolic pathways remains a question for further investigation.

Vitamins are required for healthy growth and development. The roles of vitamins against viral infection have also been reported.^8^ For example, vitamins C and D have been suggested to exert anti-COVID-19 activity.^9^ Vitamin A (also called retinol; ROL) is a generic term for many related compounds. ROL is converted to retinoic acid (RA), which in turn affects gene transcription.^10^^,^^11^ Three main steps are involved in RA synthesis.^12^ ROL is oxidized by retinol dehydrogenase 10 (RDH10) to retinol aldehyde (RAL), which is further oxidized by retinol dehydrogenase (RALDH) to generate RA. Additionally, RAL can be reduced to ROL by retinol reductase 3 (DHRS3). Although RA exhibits antiviral effects against viral infection,^13^ whether and how viruses reprogram RA metabolism is currently unknown.

A close relationship between the immune system and host metabolism has been known for decades. For instance, lactate acts as a natural suppressor of RIG-I-like receptor signaling by targeting mitochondrial antiviral-signaling protein.^14^ Serine metabolism antagonizes antiviral innate immunity by preventing ATP6V0d2-mediated YAP lysosomal degradation.^15^ Hexokinase 2 and glycolysis-derived lactate have important functions in the immune escape of hepatitis B virus (HBV), and energy metabolism regulates innate immunity during HBV infection.^16^ However, whether and how other host metabolic factors influence antiviral innate immunity remains largely unknown.

In this study, we unexpectedly found that viral infection inhibited RA synthesis, thereby facilitating viral replication. Mechanistic studies revealed that viral infection inhibited RA synthesis through P53-mediated upregulation of DHRS3, thereby preventing lipid efflux and immune activation.

## Results

### RA synthesis is inhibited by viral infection *in vitro* and *in vivo*

**Figure 1:**
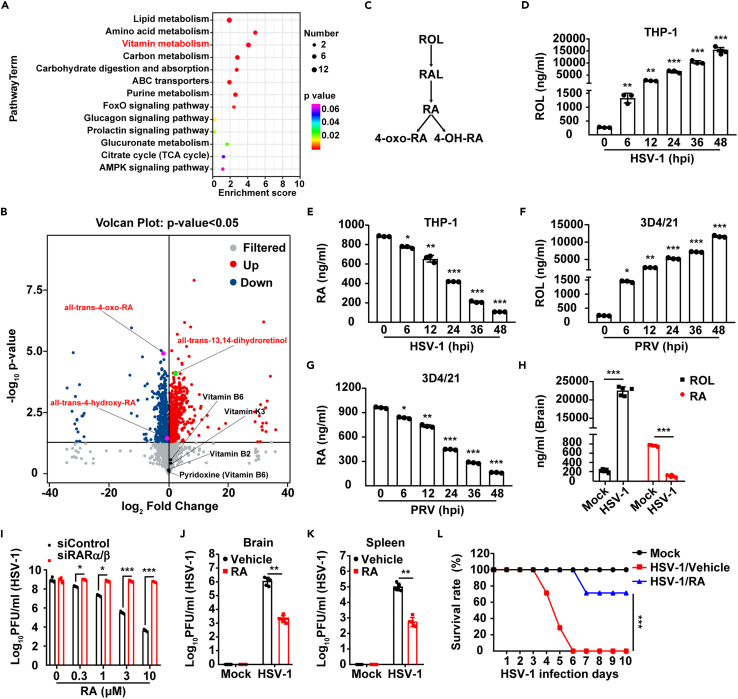
Viral infection inhibits RA synthesis and facilitates viral proliferation (A) KEGG-enriched metabolic pathways of THP-1 cells mock-infected or infected with HSV-1 (MOI = 0.1) at 48 hpi. (B) Volcano plot showing the differential metabolites from (A). Spots representing vitamins and their metabolites are indicated. (C) Schematic diagram of RA metabolism. (D and E) HPLC analysis of ROL (D) and RA (E) in THP-1 cells infected with HSV-1 (MOI = 0.1) at the indicated times. (F and G) HPLC analysis of ROL (F) and RA (G) in 3D4/21 cells infected with PRV-QXX (MOI = 1) at the indicated times. (H) HPLC analysis of ROL and RA in the brain in mice mock-infected or infected with HSV-1 (1 × 10^6^ pfu per mouse) at 5 days post-infection (*n* = 5). (I) Viral titer analysis in siControl and siRARα/β THP-1 cells infected with HSV-1 (MOI = 0.1) combined with treatment with RA (0–10 μM) for 24 h. (J and K) Viral titer analysis in the brain (J) and spleen (K) in mice mock-infected or infected with HSV-1 (1 × 10^6^ pfu per mouse) combined with treatment with vehicle or RA (2.5 mg/kg) at 5 days post-infection (*n* = 5). (L) Survival rate of mice mock-infected or infected with HSV-1 (1 × 10^7^ pfu per mouse) combined with treatment with vehicle or RA (2.5 mg/kg) for 10 days (*n* = 12). Data are expressed as mean ± SD of 3 independent experiments. *p* values were determined by Student’s t test, ^∗^*p* < 0.05, ^∗∗^*p* < 0.01, ^∗∗∗^*p* < 0.001.

To investigate the delicate interplay between metabolic changes and viral infection, we used an unbiased metabolomics approach to identify global metabolic changes during herpes simplex virus type 1 (HSV-1) infection in THP-1 cells. Lipid metabolism showed significant changes following HSV-1 infection (Figure 1A), consistent with the heightened *de novo* phospholipid synthesis observed post-infection with HSV-1.^17^ Notably, we identified three differentially present categories of vitamin metabolites of ROL, vitamin B, and vitamin K (Figures 1A and 1B): except for the metabolites of vitamin B and vitamin K, only ROL and its metabolites were significantly altered in response to HSV-1 infection (Figure 1B; Figure S1A). All-trans-13,14-dihydroretinol increased, and all-trans-4-oxo-RA and all-trans-4-hydroxy-RA decreased after viral infection (Figures 1B and 1C; Figure S1A), thus suggesting that HSV-1 inhibited RA synthesis from ROL.

We next verified the alterations in ROL (all-trans-13,14-dihydroretinol) and RA (all-trans-RA) by high-performance liquid chromatography (HPLC). THP-1 cells were infected with HSV-1, and cell extracts were prepared at 0–48 hpi. HPLC analysis indicated an increase in ROL and decrease in RA (Figures 1D and 1E; Figure S1B). Consistent results were also obtained in 3D4/21 cells infected with PRV, a porcine alphaherpesvirus^18^ (Figures 1F and 1G; Figure S1C). The expression of RA-regulated genes, such as *RARA*, *RARB*, *CRABP1*, *HOXB1*, and *STRA6*, were all downregulated after HSV-1 infection (Figures S1D–S1H), thus further demonstrating that RA synthesis was inhibited by viral infection. To determine whether viral infection influenced RA metabolism *in vivo*, we mock-infected or intranasally infected mice with HSV-1. HPLC analysis showed that more ROL and less RA was detected in HSV-1-infected than mock-infected mouse brains at 5 days post-infection (Figure 1H). Together, the results suggest that viral infection inhibits RA synthesis.

We hypothesized that viral infection might inhibit RA synthesis, thus facilitating viral replication. Therefore, we introduced RA to HSV-1-infected THP-1 cells and detected that HSV-1 replication was declined in an RA dose dependent manner (Figure 1I). However, RA did not inhibit HSV-1 replication in RARα/β knockdown THP-1 cells (Figure 1I; Figure S1I); this suggests that RA affects HSV-1 replication in a receptor-dependent manner. In addition, HPLC analysis detected comparable amounts of RA in the brain in mock-infected mice and HSV-1-infected mice injected with RA at 2.5 mg/kg (Figure S1J). Restoration of RA significantly inhibited HSV-1 replication in the mouse brain and spleen (Figures 1J and 1K), as well as HSV-1-induced mortality (Figure 1L). Together these results demonstrate that viral infection prevents RA synthesis for optimal replication.

### Virus-induced upregulation of DHRS3 is responsible for the decreased RA synthesis

**Figure 2:**
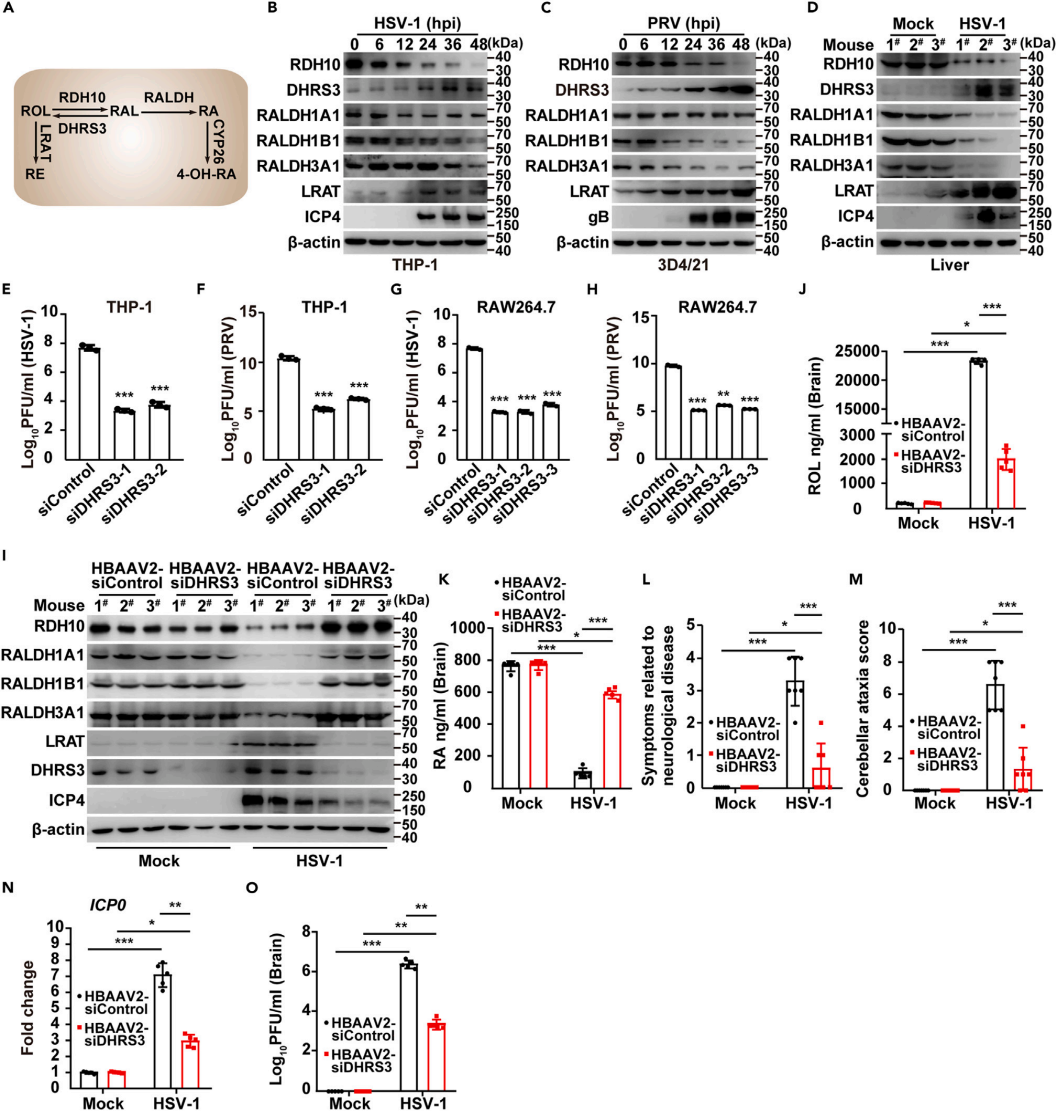
Viral infection upregulates DHRS3 and inhibits RA synthesis (A) Schematic diagram of RA metabolic pathways. (B) Immunoblotting of the indicated proteins in THP-1 cells infected with HSV-1 (MOI = 0.1) at the indicated times. (C) Immunoblotting of the indicated proteins in 3D4/21 cells infected with PRV-QXX (MOI = 1) at the indicated times. (D) Immunoblotting of the indicated proteins in the liver in mice mock-infected or infected with HSV-1 (1 × 10^6^ pfu per mouse) at 5 days post-infection (*n* = 3). (E and F) Viral titer analysis in siControl or siDHRS3 THP-1 cells infected with HSV-1 (E, MOI = 0.1) or PRV-QXX (F, MOI = 1) at 24 hpi. (G and H) Viral titer analysis in siControl or siDHRS3 RAW264.7 cells infected with HSV-1 (G, MOI = 1) or PRV-QXX (H, MOI = 1) at 24 hpi. (I) Immunoblotting of the indicated proteins in the brain in mice injected with HBAAV2-siControl (1.3 × 10^11^ vg per mouse) or HBAAV2-siDHRS3 (1.3 × 10^11^ vg per mouse) for 28 days, then mock-infected or infected with HSV-1 (1 × 10^6^ pfu per mouse) at 6 days post-infection (*n* = 3). (J and K) HPLC analysis of ROL (J) and RA (K) in the brain in mice from (I) (*n* = 5). (L) Symptoms associated with neurological disease in mice injected with HBAAV2-siControl (1.3 × 10^11^ vg per mouse) or HBAAV2-siDHRS3 (1.3 × 10^11^ vg per mouse) for 28 days and then mock-infected or infected with HSV-1 (1 × 10^6^ pfu per mouse) at 4 days post-infection (*n* = 7). (M) Cerebellar ataxia scores of mice from (L) (*n* = 7). (N) RT-qPCR analysis of HSV-1 *ICP0* in the trigeminal nerve of mice from (I) (*n* = 5). (O) Viral titer analysis in the brain of mice from (I) (*n* = 5). Data are expressed as mean ± SD of 3 independent experiments. *p* values were determined by Student’s t test, ^∗^*p* < 0.05, ^∗∗^*p* < 0.01, ^∗∗∗^*p* < 0.001.

Three main steps are involved in RA metabolism (Figure 2A). To determine how viral infection inhibited RA synthesis, we first analyzed the expression of the enzymes involved in RA metabolism. Immunoblotting indicated that viral infection downregulated retinol dehydrogenase 10 (RDH10) and retinaldehyde dehydrogenase (RALDH), and upregulated retinaldehyde reductase (DHRS3) and lecithin retinol acyl transferase (LRAT), as indicated in THP-1, bone marrow-derived macrophages (BMDMs), and 3D4/21 cells (Figures 2B and 2C; Figure S2A), as well as in mouse liver (Figure 2D). RDH10 expression correlates with the expression of RARα;^19^ therefore, RDH10 expression may be regulated by RA. DHRS3 is an RA-induced gene.^20^ We explored the transcripts of *Rdh10* and *Dhrs3*. As expected, the mRNA level of *Rdh10* was downregulated in HSV-1-infected RAW264.7 cells and PRV-infected 3D4/21 cells (Figures S2B and S2C). Unexpectedly, *Dhrs3* transcription was upregulated by viral infection (Figures S2B and S2C). Because we observed that RA was declined in response to viral infection, these data indicated that viral infection prevented RA synthesis through upregulation of DHRS3.

We then knocked down DHRS3 expression in THP-1 and RAW264.7 cells with small interfering RNA (siRNA). Immunoblotting indicated that knockdown of DHRS3 inhibited the expression of HSV-1 ICP4 and PRV gB (Figures S2D and S2E). In addition, plaque assays demonstrated that knockdown of DHRS3 expression affected the replication of HSV-1 and PRV (Figures 2E–2H). We further used HBAAV2-mediated DHRS3 knockdown *in vivo* (Figure S2F). HBAAV2 can cross the blood-brain barrier and HBAAV2-mediated DHRS3 knockdown was effective in mouse brain, as well as the spleen and liver (Figure S2G). Interference of DHRS3 in the HSV-1-infected mouse brain restored the expression of RDH10, RALDH, and LRAT to the control levels (Figure 2I). After HSV-1 infection, ROL was lower, and RA was higher, in the brain of mice injected with HBAAV2-siDHRS3 than in HBAAV2-siControl injected mice (Figures 2J and 2K). We observed attenuated disease development in mice with DHRS3 knockdown, as shown by disease scores reflecting central nervous system infection (Figures 2L and 2M). Moreover, decreased mRNA levels of HSV-1 *ICP0* were detected in the trigeminal nerves in HBAAV2-siDHRS3-injected mice (Figure 2N), and knockdown of DHRS3 suppressed HSV-1 replication in the brain and spleen (Figure 2O; Figure S2H). These data suggest that viral infection prevents RA synthesis through upregulation of DHRS3, thus facilitating viral replication.

### P53 is responsible for virus-induced DHRS3 upregulation

**Figure 3:**
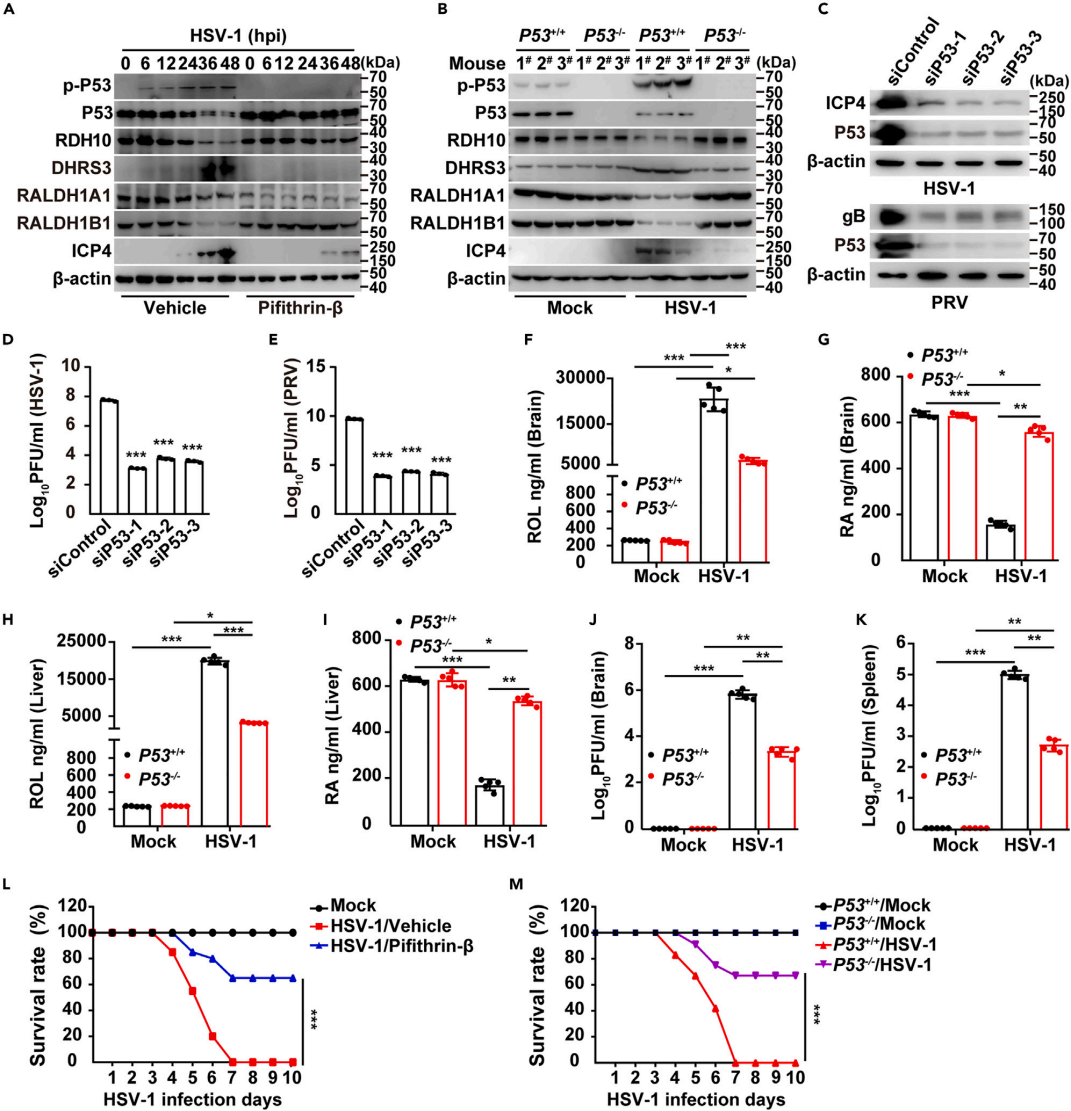
Viral infection stimulates DHRS3 expression through P53 activation (A) Immunoblotting of the indicated proteins in THP-1 cells infected with HSV-1 (MOI = 0.1) combined with treatment with vehicle or pifithrin-β (10 μM) for the indicated times. (B) Immunoblotting of the indicated proteins in the brain in *P53*^*+/+*^ and *P53*^*−/−*^ mice mock-infected or infected with HSV-1 (1 × 10^6^ pfu per mouse) at 5 days post-infection (*n* = 3). (C) Immunoblotting of the indicated proteins in siControl or siP53 THP-1 cells infected with HSV-1 (MOI = 0.1) or PRV-QXX (MOI = 1) at 24 hpi. (D and E) Viral titer assay of siControl or siP53 THP-1 cells infected with HSV-1 (D, MOI = 0.1) or PRV-QXX (E, MOI = 1) at 24 hpi. (F–I) HPLC analysis of ROL (F and H) and RA (G and I) in the brain (F and G) and liver (H and I) in *P53*^*+/+*^ and *P53*^*−/−*^ mice from (B) (*n* = 5). (J and K) Viral titer assay in the brain and spleen in *P53*^*+/+*^ and *P53*^*−/−*^ mice from (B) (*n* = 5). (L) Survival rate of mice mock-infected or infected with HSV-1 (1 × 10^7^ pfu per mouse) combined with treatment with vehicle or pifithrin-β (22 mg/kg) for 10 days (*n* = 12). (M) Survival rate of *P53*^*+/+*^ and *P53*^*−/−*^ mice mock-infected or infected with HSV-1 (1 × 10^7^ pfu per mouse) for 10 days (*n* = 12). Data are expressed as mean ± SD of 3 independent experiments. *p* values were determined by Student’s t test, ^∗^*p* < 0.05, ^∗∗^*p* < 0.01, ^∗∗∗^*p* < 0.001.

To investigate the molecular mechanism through which viral infection stimulated DHRS3 expression, we first analyzed the regulatory elements in the *DHRS3* promoter and found four potential P53 binding sites (Figure S3A). P53 is essential for the replication of HSV-1.^21^ Immunoblotting indicated that both HSV-1 and PRV enhanced phosphorylated P53 (p-P53), and the P53 downstream effectors MDM2 and P21 (Figures S3B and S3C), thus indicating that viral infection activated P53. Pifithrin-β is an inhibitor of the P53 protein.^22^ Treatment of THP-1 cells with pifithrin-β resulted in the inhibition of HSV-1-induced upregulation of DHRS3 and restoration of RDH10 and RALDH expression levels (Figure 3A). Consistent results were obtained from *p53* knockout mice (Figure 3B). Knockdown of P53 significantly affected viral replication, as indicated by immunoblotting for HSV-1 ICP4 and PRV gB (Figure 3C), and plaque assays of viral titer (Figures 3D and 3E). The transcription of *Dhrs3* was defective in the brains of *P53*^−/−^ mice in response to HSV-1 infection (Figure S3D). These results suggest that viral infection upregulates DHRS3 expression through P53 activation.

We next explored the role of P53 in RA metabolism *in vivo*. HPLC analysis indicated that lower ROL and higher RA were induced by HSV-1 infection in the brain and liver in *P53*^−/−^ mice than *P53*^+/+^ mice (Figures 3F–3I), thus suggesting that viral infection reprogramed RA metabolism through P53-regulated DHRS3 expression. We also examined HSV-1 replication in control and *P53* knockout mice. Plaque assays indicated that knockout of P53 significantly suppressed HSV-1 replication in the mouse brain and spleen (Figures 3J and 3K). Furthermore, the HSV-1-challenged mice all died at 7 days post-infection in the control group (Figure 3L). However, approximately 60% of mice injected with pifithrin-β survived until 10 days post-infection (Figure 3L). The survival rate was also higher in *P53*^−/−^ mice than *P53*^+/+^ mice at 10 days after HSV-1 infection (Figure 3M). All the data indicate that viral infection promotes P53-dependent DHRS3 upregulation, thereby inhibiting RA synthesis.

### Viral infection promotes downregulation of HDAC1/2 and hyperacetylation of histones H3 and H4

**Figure 4:**
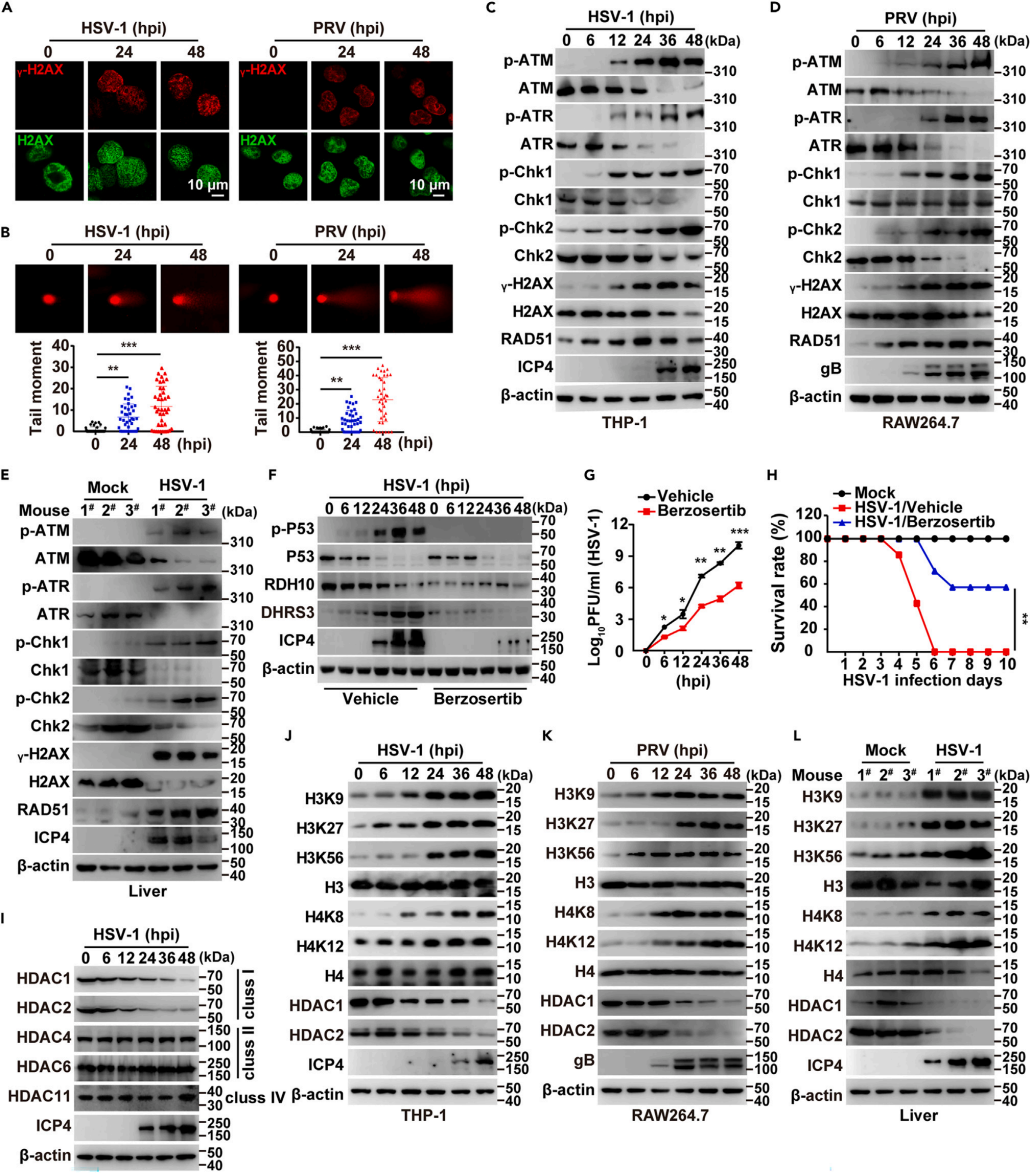
Viral infection downregulated the expression of class I HDACs and caused hyperacetylation of histones H3 and H4 (A) Immunofluorescence of γ-H2AX and H2AX in THP-1 cells infected with HSV-1 (MOI = 0.1) or 3D4/21 cells infected with PRV (MOI = 1) for the indicated times. Scale bar, 10 μm. (B) Comet assay of DNA damage in THP-1 cells infected with HSV-1 (MOI = 0.1) or 3D4/21 cells infected with PRV (MOI = 1) for the indicated times. (C) Immunoblotting of the indicated proteins in THP-1 cells infected with HSV-1 (MOI = 0.1) for the indicated times. (D) Immunoblotting of the indicated proteins in RAW264.7 cells infected with PRV-QXX (MOI = 1) for the indicated times. (E) Immunoblotting of the indicated proteins in the liver in mice mock-infected or infected with HSV-1 (1 × 10^6^ pfu per mouse) at 5 days post-infection (*n* = 3). (F) Immunoblotting of the indicated proteins in THP-1 cells infected with HSV-1 (MOI = 0.1) combined with treatment with vehicle or berzosertib (50 nM) for the indicated times. (G) Viral titer analysis in THP-1 cells from (F). (H) Survival rate of mice mock-infected or infected with HSV-1 (1 × 10^7^ pfu per mouse) combined with treatment with vehicle or berzosertib (20 mg/kg) for 10 days (*n* = 12). (I and J) Immunoblotting of the indicated proteins in THP-1 cells from (C). (K) Immunoblotting of the indicated proteins in RAW264.7 cells from (D). (L) Immunoblotting of the indicated proteins in the liver in mice from (E) (*n* = 3). Data are expressed as mean ± SD of 3 independent experiments. *p* values were determined by Student’s t test, ^∗^*p* < 0.05, ^∗∗^*p* < 0.01, ^∗∗∗^*p* < 0.001.

P53 is a transcription factor highly inducible by many stress signals, such as the DNA damage response (DDR), oncogene activation and nutrient deprivation.^23^ Because alphaherpesvirus induces DDR,^24^^,^^25^^,^^26^ we hypothesized that DDR might be responsible for HSV-1-induced P53 activation and subsequent DHRS3 upregulation. Indeed, viral infection resulted in DDR, as indicated by immunofluorescence of phosphorylated H2AX (γ-H2AX), the most sensitive marker of DDR,^27^ and by comet assays of DNA double-strand breaks^28^ (Figures 4A and 4B). When DDR occurs, cells must repair damaged DNA to maintain the integrity of their genomes.^29^ We then investigated DNA lesion-activated checkpoint pathways. Immunoblotting indicated that HSV-1 infection enhanced the phosphorylation of ATM, ATR, Chk1, Chk2, H2AX, and RAD51 in THP-1, BMDM, and mouse liver (Figures 4C–4E; Figure S4A). These results suggest that viral infection triggers DDR.

To determine the roles of DDR in RA metabolism and viral infection, we inhibited DDR with berzosertib.^30^ HSV-1-induced transcription of *DHRS3* was inhibited by berzosertib treatment (Figure S4B). Immunoblotting indicated that treatment of THP-1 cells with berzosertib prevented P53 phosphorylation and DHRS3 upregulation during HSV-1 infection (Figure 4F). Meanwhile, RDH10 expression was unaltered (Figure 4F). Plaque assays of viral titer showed that HSV-1 replication decreased in THP-1 cells under berzosertib treatment (Figure 4G). As compared with vehicle-injected mice, approximately 60% of HSV-1-infected mice survived after berzosertib injection (Figure 4H). Simultaneously, the virus titer plaque assay results indicated a decrease in HSV-1 replication in siCDC25c cells (Figure S4C). These results indicate that DDR is beneficial for viral replication.

The post-translational modification of histone acetylation is a key mechanism that regulates chromatin integrity.^31^ Histone deacetylases (HDACs), a family of proteins highly conserved across all eukaryotes, function by removing acetyl groups from histones.^32^ SNP rs4971059 controls a group of genes, such as those essential for DNA replication and repair, by increasing TRIM46 expression, which in turn targets HDAC1 for ubiquitination and degradation.^33^ We explored whether viral infection might impair HDAC expression and histone acetylation. Classical HDAC proteins are categorized into class I (HDAC1, HDAC2, HDAC3, and HDAC8), class II (HDAC4, HDAC5, HDAC6, HDAC7, HDAC9, and HDAC10), and class IV (HDAC11).^34^ Intriguingly, we observed that expressions of HDAC1 and HDAC2 were decreased after HSV-1 infection, whereas those of HDAC4, HDAC6, and HDAC11 were not affected (Figure 4I). These results suggested that HSV-1 specifically decreased class I HDACs. We then analyzed whether histone acetylation was enhanced by viral infection. Immunoblotting of histones H3 and H4 with specific acetylated antibodies showed that acetyl groups in lysines 9, 27, and 56 of histone H3 (H3K9, H3K27, and H3K56) and lysines 8 and 12 of histone H4 (H4K8 and H4K12) were increased during HSV-1 infection in THP-1 cells and in BMDM (Figure 4J; Figure S4D), as well as in PRV-infected RAW264.7 cells (Figure 4K). Furthermore, acetylation of histones H3 and H4 was also enhanced in HSV-1-infected mouse liver (Figure 4L). Moreover, we discovered that blocking HDAC1/2 resulted in increased acetylation of H3 and H4, leading to the activation of DDR downstream signaling pathways following treatment with HDAC1 inhibitors (Figure S4E). Trichostatin A (TSA) is a potent inhibitor of HDAC class I and II (HDAC class I/II), showing high specificity and effectiveness.^35^ Together, these results demonstrate that viral infection induces DDR through a decrease in class I HDACs, and subsequent hyperacetylation of histones H3 and H4.

### Viral infection inhibits RA synthesis, thereby preventing lipid efflux

**Figure 5:**
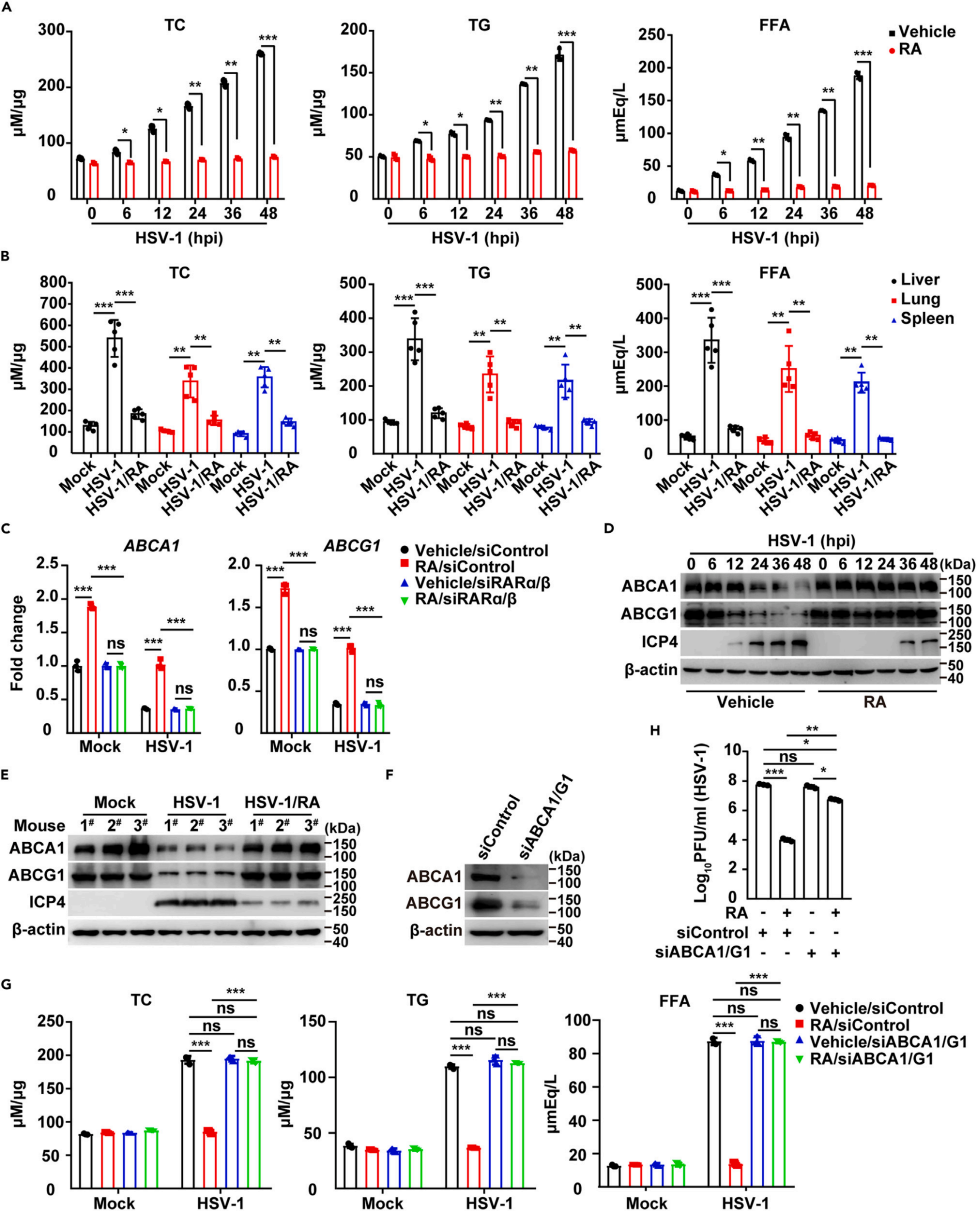
Viral infection inhibits RA synthesis to prevent lipid efflux (A) TC, TG, and FFA in THP-1 cells infected with HSV-1 (MOI = 0.1) combined with treatment with vehicle or RA (10 μM) for the indicated times. (B) TC, TG, and FFA in the liver, lung, and spleen in mice mock-infected or infected with HSV-1 (1 × 10^6^ pfu per mouse) combined with treatment with vehicle or RA (2.5 mg/kg) for 5 days (*n* = 5). (C) RT-qPCR analysis of *ABCA1* and *ABCG1* in siControl and siRARα/β THP-1 cells mock-infected or infected with HSV-1 (MOI = 0.1) combined with treatment with vehicle or RA (10 μM) for 24 h. (D) Immunoblotting of the indicated proteins in THP-1 cells infected with HSV-1 (MOI = 0.1) combined with treatment with vehicle or RA (10 μM) for the indicated times. (E) Immunoblotting of the indicated proteins in the liver in mice from (B) (*n* = 3). (F) Immunoblotting of the indicated proteins in siControl and siABCA1/G1 THP-1 cells. (G) TC, TG, and FFA in siControl and siABCA1/G1 THP-1 cells mock-infected or infected with HSV-1 (MOI = 0.1) combined with treatment with vehicle or RA (10 μM) for 24 h. (H) Viral titer analysis in siControl and siABCA1/G1 THP-1 cells infected with HSV-1 (MOI = 0.1) combined with treatment with vehicle or RA (10 μM) for 24 h. Data are expressed as mean ± SD of 3 independent experiments. *p* values were determined by Student’s t test, ^∗∗^*p* < 0.01, ^∗∗∗^*p* < 0.001. ns, no significance.

Lipid metabolism modulates viral infection by participating in the viral replication cycle.^36^^,^^37^ Additionally, it has been discovered that HSV-1 replication and pathogenicity depend on the biosynthesis of phosphatidylethanolamine (PE) facilitated by phosphate cytidylyltransferase 2-ethanolamine (Pcyt2).^38^ The KEGG-enriched metabolic pathways indicated that lipid metabolism was significantly altered after HSV-1 infection (Figure 1A). Twelve metabolites contributing to lipid metabolism were all increased after HSV-1 infection (Figure S5A). Lipid quantification indicated that the virus-induced increases in total cholesterol (TC), triglycerides (TG), and free fatty acids (FFA) in THP-1 cells and in mouse tissues were inhibited by RA (Figures 5A and 5B). In addition, treatment of THP-1 cells with pifithrin-β and berzosertib inhibited the virus-induced increase in TC, TG, and FFA (Figures S5B and S5C), thus indicating that P53 and DDR were responsible for the increase in lipids.

We then examined the roles of RA in lipid metabolism. We first analyzed the transcription of the key enzymes in lipid synthesis, lipolysis and lipid efflux.^39^ The RT-qPCR and immunoblotting indicated that viral infection upregulated lipid synthesis enzymes, such as HMGCR, SREBP2, FASN, and ACC (Figures S5D and S5F). Supplementation with RA had no effects on their expression (Figures S5D and S5F), thus suggesting that RA did not play roles in lipid synthesis. Adipose triglyceride lipase (ATGL), Monoacyl glycerol lipase (MGLL), and Hormone-sensitive triglyceride lipase (HSL), which are involved in lipolysis, were stimulated by viral infection (Figures S5E and S5F). However, they were downregulated by RA (Figures S5E and S5F). This result suggested that RA inhibited virus-induced lipolysis. ATP-binding cassette (ABC) transporters are integral membrane proteins responsible for translocation of many substrates across membranes.^40^ *ABCB1*, *ABCC1*, and *ABCD1* were all upregulated during viral infection and downregulated by RA (Figure S5G). ABCA1 and ABCG1 are important transporters mediating lipid efflux.^41^ RT-qPCR and immunoblotting indicated that HSV-1 decreased both the mRNA and protein levels of ABCA1 and ABCG1, whereas this decrease was restored by RA supplementation (Figures 5C–5E), thus suggesting that viral infection inhibited RA synthesis and consequently prevented ABCA1/G1-mediated lipid efflux.

To better confirm the role of RA in lipid efflux, we inhibited ABCA1/G1 expression through RNA interference in THP-1 cells (Figure 5F). In response to viral infection, comparable TC, TG, and FFA were detected in vehicle-treated control cells with vehicle-treated and RA-treated cells with ABCA1/G1 knockdown (Figure 5G), thereby further suggesting that viral infection inhibited RA synthesis and consequently prevented ABCA1/G1-mediated lipid efflux. The plaque assay results indicated that the presence of RA led to a partial inhibition of HSV-1 replication in si ABCA1/G1 THP-1 cells (Figure 5H). This could be attributed to the fact that siRNA knockdown does not completely eliminate protein expression, resulting in some residual protein that may account for the observed slight effect. These findings suggest that viral infection hinders RA synthesis, consequently impeding ABCA1/G1-mediated lipid efflux to some extent.

### Viral infection inhibits RA synthesis and promotes evasion of antiviral innate immunity

**Figure 6:**
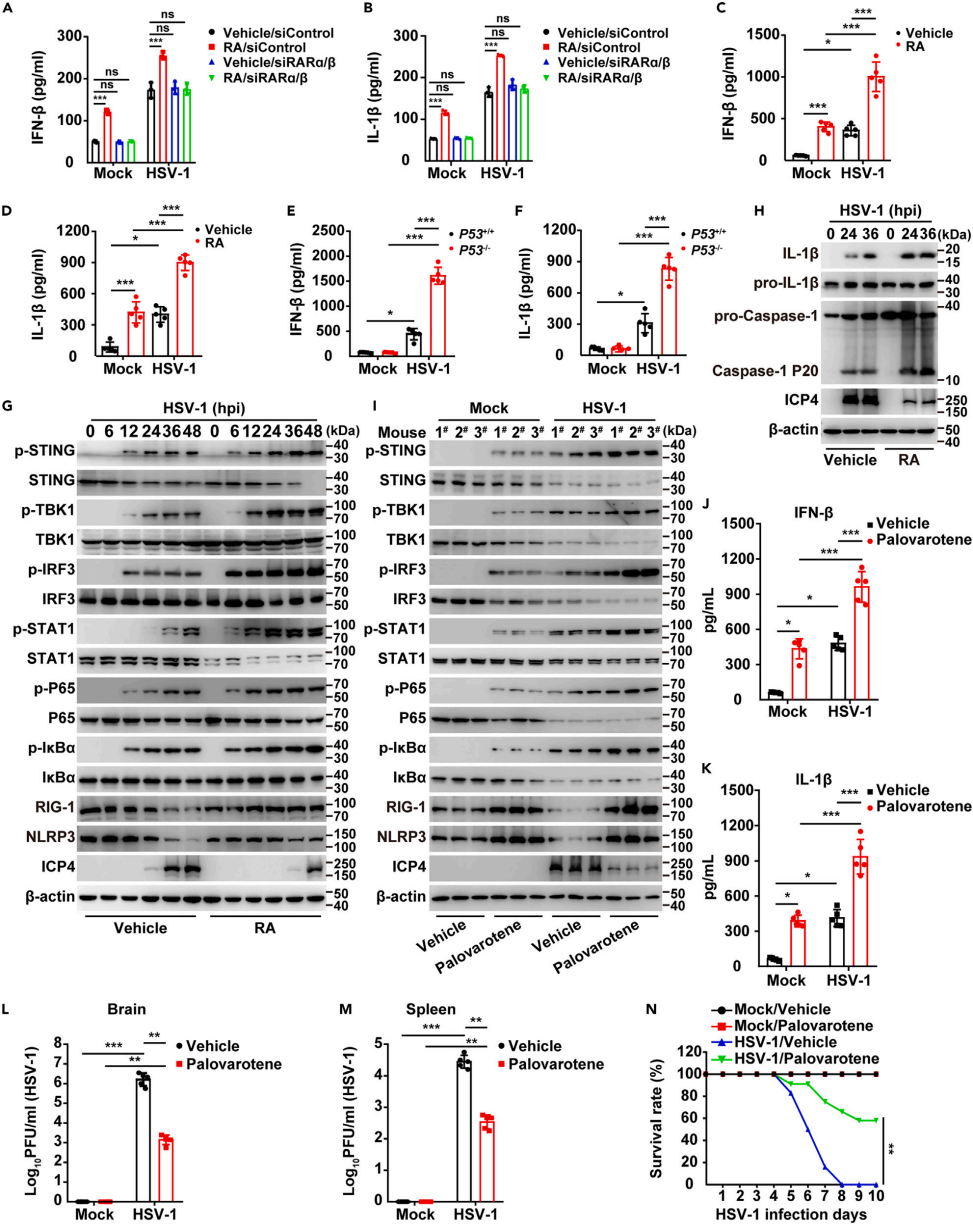
Viral infection inhibits RA synthesis to prevent the innate immune response (A and B) IFN-β (A) and IL-1β (B) secretion from siControl or siRARα/β THP-1 cells mock-infected or infected with HSV-1 (MOI = 0.1) combined with treatment with vehicle or RA (10 μM) for 24 h. (C and D) IFN-β (C) and IL-1β (D) in the serum in mice mock-infected or infected with HSV-1 (1 × 10^6^ pfu per mouse) combined with treatment with vehicle or RA (2.5 mg/kg) for 5 days. (E and F) IFN-β (E) and IL-1β (F) in the serum of *P53*^*+/+*^ and *P53*^*−/−*^ mice mock-infected or infected with HSV-1 (1 × 10^6^ pfu per mouse) for 5 days. (G) Immunoblotting of the indicated proteins in THP-1 cells infected with HSV-1 (MOI = 0.1) combined with treatment with vehicle or RA (10 μM) for 0–48 h. (H) Immunoblotting of the indicated proteins in THP-1 cells infected with HSV-1 (MOI = 0.1) combined with treatment with vehicle or RA (10 μM) for 0–36 h. (I) Immunoblotting of the indicated proteins in the liver in mice mock-infected or infected with HSV-1 (1 × 10^6^ pfu per mouse) combined with treatment with vehicle or palovarotene (1 mg/kg) for 5 days (*n* = 3). (J and K) IFN-β (J) and IL-1β (K) in the serum in mice from (I) (*n* = 5). (L and M) Viral titer analysis in the brain (L) and spleen (M) in mice from (I) (*n* = 5). (N) Survival rate in mice mock-infected or infected with HSV-1 (1 × 10^7^ pfu per mouse) combined with treatment with vehicle or palovarotene (1 mg/kg) for 10 days (*n* = 12). Data are expressed as mean ± SD of 3 independent experiments. *p* values were determined by Student’s t test, ^∗^*p* < 0.05, ^∗∗^*p* < 0.01, ^∗∗∗^*p* < 0.001.

Intriguingly, RA supplementation inhibited HSV-1 replication in siABCA1/G1 THP-1 cells (Figure 5H), thus suggesting the existence of other potential mechanisms. Because RA affected HSV-1 replication in a receptor-dependent manner (Figure 1I), we performed RNA sequencing (RNA-seq) in THP-1 cells to explore RA-regulated genes involved in the inhibition of viral replication. KEGG-enriched pathways indicated that immune responses were significantly stimulated by RA (Figures S6A–S6E). Therefore, we hypothesized that viral infection might inhibit RA synthesis and support evasion of antiviral innate immunity. As shown in Figures 6A and 6B, IFN-β and IL-1β secretion was promoted by RA or by HSV-1 infection in control cells. RA further stimulated virus-induced IFN-β and IL-1β secretion, which was abrogated in siRARα/β THP-1 cells (Figures 6A and 6B). A similar result was also observed in mouse serum (Figures 6C and 6D). Furthermore, higher serum IFN-β and IL-1β were detected in *p53*^*−/−*^ mice than in *p53*^*+/+*^ mice under HSV-1 challenge (Figures 6E and 6F). Inhibition of P53, DHRS3, and DDR in THP-1 cells also upregulated IFN-β and IL-1β secretion in response to HSV-1 infection (Figures S6F–S6K). We then analyzed the activation of innate immune pathways. Immunoblotting indicated that RA enhanced the phosphorylation of STING, TBK1, IRF3, STAT1, P65, and IκBα, and the expression of RIG-1 and NLRP3 (Figures 6G and S6K). Inflammasomes were also enhanced by viral infection, as indicated by immunoblotting for mature IL-1β and cleaved caspase-1 P20 (Figure 6H). These data indicate that viral infection inhibits RA synthesis, thereby facilitating evasion of antiviral innate immunity.

Palovarotene is an orally bioavailable selective RA receptor agonist being developed by Ipsen for decreasing the formation of heterotopic ossification in patients with fibrodysplasia ossificans progressiva.^42^ Since RARα, RARβ, and RARγ belong to the nuclear receptor (NR) transcription factor superfamily and can form heterodimers with members of the retinoid X receptor (RXR) subfamily, palovarotene, an agonist of the RA gamma receptor, was assessed.^43^ We therefore tested whether palovarotene might be used as a potent antiviral agent against HSV-1. Palovarotene injection significantly activated innate immune pathways in mock-infected and HSV-1-infected mice, as indicated by immunoblotting of phosphorylated STING, TBK1, IRF3, STAT1, P65, and IκBα, and the expression of RIG-1 and NLRP3 (Figure 6I). More IFN-β and IL-1β were detected in palovarotene-injected mouse serum than that in control mice (Figures 6J and 6K). Plaque assays of viral titer indicated that palovarotene inhibited HSV-1 replication in the mouse brain and spleen (Figures 6L and 6M). The survival rate was higher in palovarotene-injected mice than vehicle-injected mice 10 days after HSV-1 infection (Figure 6N). These results suggested that palovarotene had antiviral activity against HSV-1.

## Discussion

**Figure 7:**
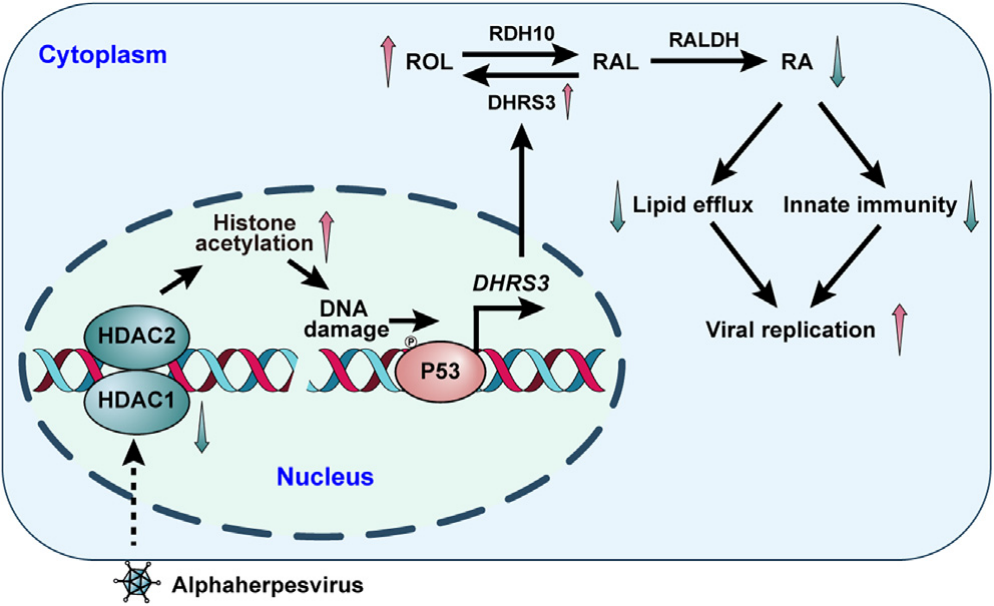
A schematic model showing alphaherpesvirus down-regulates RA to facilitate viral replication

HSV-1 is a highly abundant human pathogen with worldwide prevalence levels of approximately 67%.^44^ It causes a variety of diseases, including cold sores, genital herpes, herpes stromal keratitis, meningitis, and encephalitis.^45^ HSV-1 induces metabolic reprogramming. For example, HSV-1 infection promotes increased glucose consumption and lactate production in Vero cells.^46^ HSV-1 also upregulates pyrimidine nucleotide biosynthesis by increasing aspartate generation.^47^ Our findings revealed that HSV-1 inhibited RA synthesis, thus facilitating viral replication (Figure 7). Therefore, the development of new therapeutic approaches that circumvent HSV-1 spread through the target of RA metabolic pathways provided an opportunity to combat this infection. In this study, we evaluated the antiviral effect of the RAR agonist palovarotene and demonstrated that palovarotene significantly inhibited HSV-1 replication both in cultured cells and in a mouse model, thereby expanding the therapeutic strategies for HSV-1 infection.

Our findings and previous studies have indicated that HSV-1 induces DDR.^24^ HSV-1 UL36USP deubiquitinase suppresses DNA repair in host cells via deubiquitination of proliferating cell nuclear antigen.^48^ The ATM and ATR DNA repair pathways are activated by HSV-1 and are required for efficient viral replication.^49^ Here, we have revealed that HSV-1 promotes the downregulation of class I HDACs, thereby leading to hyperacetylation of histones H3 and H4. Hyperacetylation of H3K56 perturbs replisomes and limits break-induced replication and induction of DDR.^50^^,^^51^ These findings suggested the potent mechanisms in HSV-1-induced DDR.

P53 is a critical factor in the cellular response to a broad range of stress factors, through its ability to regulate various cellular pathways.^52^ Although P53 functions as an essential antiviral molecule against Japanese encephalitis virus,^53^ our data indicate that P53 inhibits RA synthesis through upregulation of DHRS3, thus facilitating HSV-1 replication. P53 also regulates lipid metabolism pathways.^54^ Previously, we reported that lipids were essential for PRV entry.^55^ In the present study, we demonstrated that HSV-1 upregulated lipid content both *in vitro* and *in vivo*. Our data indicated that RA did not affect the expression of lipid synthesis genes, whereas RA inhibits lipolysis and promoted lipid efflux. Therefore, these findings reveal a novel mechanism underlying how P53 regulates lipid metabolism through modulating RA synthesis.

Innate immune systems are the first line of defense against viral infection. HSV-1 infection triggers antiviral defense through the cGAS-STING pathway.^56^ How viruses evade innate immune systems is a fascinating question for virologists. Much attention has been paid to virally encoded proteins that interact with key regulators in innate immune pathways. Viruses can also evade innate immunity through metabolic reprogramming.^57^ Our findings indicated that RA activated the STING pathway and inflammasomes, and HSV-1 inhibits RA synthesis. Therefore, we suggest that HSV-1 may evade innate immunity through inhibiting RA synthesis. Recently, Maltbaek and colleagues have reported that ABCC1 exports the immunostimulatory cyclic dinucleotide cGAMP.^58^ Of note, HSV-1 infection upregulated ABCC1, which in turn can be downregulated by RA. Therefore, we suggest that HSV-1 enhanced ABCC1 expression, thus promoting cGAMP export. In addition, HSV-1 inhibited RA synthesis, thereby preventing the decline in ABCC1 induced by viral infection. This mechanism demonstrated a fresh approach to HSV-1’s immune evasion.

### Limitations of the study

One limitation of our study was that we did not identify the mechanism through which HSV-1 induced the translocation of class I HDACs into the cytoplasm. In addition, we did not elucidate how RA activated the innate immune response. A more detailed analysis of the molecular basis of this process would aid in further understanding the relationship between RA-regulated immunity and infectious diseases.

## STAR★Methods

### Key resources table

**Table:** 

REAGENT or RESOURCE	SOURCE	IDENTIFIER
**Antibodies**		

Rabbit Polyclonal anti-RARα	Proteintech	Cat# 10331-1-AP, RRID: AB_2177742
Rabbit Polyclonal anti-RARβ	Proteintech	Cat# 14013-1-AP, RRID: AB_2177765
Rabbit polyclonal anti-RDH10	Proteintech	Cat# 14644-1-AP RRID: AB_2177866
Rabbit polyclonal anti-DHRS3	Proteintech	Cat# 15393-1-AP RRID: AB_2091855
Rabbit polyclonal anti-ALDH1A1	Proteintech	Cat# 15910-1-AP RRID: AB_2305276
Rabbit polyclonal anti-ALDH1B1	Proteintech	Cat# 15560-1-AP RRID: AB_2224162
Rabbit polyclonal anti-ALDH3A1	Proteintech	Cat# 15578-1-AP RRID: AB_2273745
Rabbit polyclonal anti-LRAT	Proteintech	Cat# 12815-1-AP RRID: AB_2297180
Rabbit polyclonal anti-P53	Proteintech	Cat# 10442-1-AP RRID: AB_2206609
Rabbit polyclonal anti-CHK1	Proteintech	Cat# 25887-1-AP RRID: AB_2880283
Rabbit polyclonal anti-CHK2	Proteintech	Cat# 13954-1-AP RRID: AB_10598159
Rabbit polyclonal anti-RAD51	Proteintech	Cat# 14961-1-AP RRID: AB_2177083
Mouse monoclonal anti-β-actin	Proteintech	Cat# 66009-1-lg RRID: AB_2782959
Rabbit polyclonal anti-HDAC1	Proteintech	Cat# 10197-1-AP RRID: AB_2118062
Rabbit polyclonal anti-HDAC2	Proteintech	Cat# 12922-3-AP RRID: AB_2118516
Rabbit polyclonal anti-HDAC4	Proteintech	Cat# 17449-1-AP RRID: AB_2118864
Rabbit polyclonal anti-HDAC6	Proteintech	Cat# 12834-1-AP RRID: AB_10597094
Mouse monoclonal anti-HDAC11	Proteintech	Cat# 67949-1-Ig, RRID:AB_2918701
Rabbit polyclonal anti-ABCG1	Proteintech	Cat# 13578-1-AP, RRID: AB_2220174
Rabbit polyclonal anti-STING	Proteintech	Cat# 19851-1-AP RRID: AB_10665370
Rabbit polyclonal anti-RIG1	Proteintech	Cat# 20566-1-AP RRID: AB_10700006
Rabbit polyclonal anti-NLRP3	Proteintech	Cat# 19771-1-AP RRID: AB_10646484
Rabbit polyclonal anti-P21	Proteintech	Cat# 10355-1-AP RRID: AB_2077682
Mouse monoclonal anti-p-P53	Cell Signaling Technology	Cat# 9286 RRID: AB_331741
Rabbit polyclonal anti-p-ATM	Cell Signaling Technology	Cat# 13050 RRID: AB_2798100
Rabbit polyclonal anti-ATM	Cell Signaling Technology	Cat# 2873 RRID: AB_2062659
Rabbit polyclonal anti-p-ATR	Cell Signaling Technology	Cat# 2853 RRID:AB_2290281
Rabbit polyclonal anti-ATR	Cell Signaling Technology	Cat# 13934 RRID: AB_2798347
Rabbit polyclonal anti-p-CHK1	Cell Signaling Technology	Cat# 12302 RRID: AB_2783865
Rabbit polyclonal anti-p-CHK2	Cell Signaling Technology	Cat# 2197 RRID: AB_2080501
Mouse monoclonal anti-γ-H2AX	Cell Signaling Technology	Cat# 80312 RRID: AB_2799949
Rabbit polyclonal anti-H3	Cell Signaling Technology	Cat# 4499 RRID: AB_10544537
Rabbit polyclonal anti-H4K8ac	Cell Signaling Technology	Cat# 2594 RRID: AB_2248400
Rabbit polyclonal anti-H4K12ac	Cell Signaling Technology	Cat# 13944 RRID: AB_2798350
Rabbit polyclonal anti-H4	Cell Signaling Technology	Cat# 13919 RRID: AB_2798345
Rabbit polyclonal anti-H2AX	Cell Signaling Technology	Cat# 7631 RRID: AB_10860771
Rabbit polyclonal anti-H3K9ac	Cell Signaling Technology	Cat#4658 RRID: AB_10544405
Rabbit polyclonal anti-H3K27ac	Cell Signaling Technology	Cat# 8173 RRID: AB_10949503
Rabbit polyclonal anti-MDM2	Cell Signaling Technology	Cat# 86934 RRID: AB_2784534
Rabbit polyclonal anti-ABCA1	Cell Signaling Technology	Cat# 96292
Rabbit polyclonal anti-p-STING	Cell Signaling Technology	Cat# 50907 RRID: AB_2827656
Rabbit polyclonal anti-p-TBK1	Cell Signaling Technology	Cat# 13498 RRID: AB_2798237
Rabbit polyclonal anti-TBK1	Cell Signaling Technology	Cat# 3504 RRID: AB_2255663
Rabbit polyclonal anti-p-IRF3	Cell Signaling Technology	Cat# 29047 RRID: AB_2773013
Rabbit polyclonal anti-IRF3	Cell Signaling Technology	Cat# 11904 RRID: AB_2722521
Rabbit polyclonal anti-p-STAT1	Cell Signaling Technology	Cat# 9174 RRID: AB_2773013
Rabbit polyclonal anti-STAT1	Cell Signaling Technology	Cat# 9172 RRID: AB_2198300
Rabbit polyclonal anti-p-P65	Cell Signaling Technology	Cat# 3033 RRID: AB_331284
Rabbit polyclonal anti-P65	Cell Signaling Technology	Cat# 8242 RRID: AB_10859369
Rabbit polyclonal anti-p-IκBα	Cell Signaling Technology	Cat# 4814 RRID:AB_390781
Rabbit polyclonal anti-IκBα	Cell Signaling Technology	Cat# 7543 RRID: AB_11129663
Rabbit polyclonal anti-IL-1β	Cell Signaling Technology	Cat# 12703 RRID: AB_2737350
Rabbit polyclonal anti-Cleaved-IL-1β	Cell Signaling Technology	Cat# 83186 RRID: AB_2800010
Rabbit polyclonal anti-H3K56ac	Millipore	Cat# 07-677 RRID: AB_390167
Mouse monoclonal anti-ICP4	Abcam	Cat# ab6514 RRID: AB_305537
Mouse monoclonal anti-ICP0	Santa Cruz Biotechnology	Cat# sc-53070 RRID: AB_673704
Rabbit polyclonal anti-Caspase-1 p20	Santa Cruz Biotechnology	Cat# sc-1597 RRID: AB_2068887
Mouse monoclonal anti-FLAG	Sigma-Aldrich	Cat# F7425 RRID: AB_439687
Mouse monoclonal anti-HA	GenScript	Cat# A00169 RRID: AB_914682

**Chemicals, peptides, and recombinant proteins**		

All-trans retinoic acid	MedChemExpress	Cat# HY-14649
Pifithrin-β	MedChemExpress	Cat# HY-16702
Leptomycin B	MedChemExpress	Cat# HY-16909
Cycloheximide	MedChemExpress	Cat# HY-12320
Chloroquine	MedChemExpress	Cat# HY-17589A
MG-132	MedChemExpress	Cat# HY-13259
3-MA	MedChemExpress	Cat# HY-19312
Palovarotene	MedChemExpress	Cat# HY-14799
Phorbol 12-myristate 13-acetate	Sigma-Aldrich	Cat# 16561-29-8
DMSO	Sigma-Aldrich	Cat# W387520
ANTI-FLAG M2 affinity gel	Sigma-Aldrich	Cat# A2220
all-trans-13,14-dihydroretinol	Yuanye	Cat# B21287
Lipofectamine RNAiMAX	Invitrogen	Cat#13778075
TurboFect Transfection Reagent	Thermo Fisher Scientific	Cat# R0531
Berzosertib	Selleck	Cat# S7102

**Critical commercial assays**		

Human IL-1β ELISA Kit	R&D Systems	Cat# PDLB50
Human IFN-β ELISA Kit	R&D Systems	Cat# DIFNB0
Mouse IL-1β ELISA Kit	R&D Systems	Cat# PMLB00C
Mouse IFN-β ELISA Kit	PBL assay science	Cat# 42400

**Experimental models: Cell lines**		

Human: THP-1 Cells	ATCC	Cat# TIB-202
Human: HEK293T Cells	ATCC	Cat# CRL-11268
Human: HeLa Cells	ATCC	Cat# CL-82
Mouse: RAW264.7 Cells	ATCC	Cat# TIB-71
Mouse: L929 Cells	ATCC	Cat# CRL-6364
Sus scrofa: 3D4/21 Cells	ATCC	Cat# CRL-2843
Monkey: Vero Cells	ATCC	Cat# CL-81

**Experimental models: Organisms/strains**		

Mouse: C57BL/6N	This paper	N/A
Mouse: p53^-/-^ C57BL/6N	This paper	N/A

**Oligonucleotides**		

siRNAs and primers	Table S1	N/A

**Recombinant DNA**		

Plasmid: pEGFPC1-MDM2	This paper	N/A
Plasmid: pEGFPC1-MDM2-ΔRING	This paper	N/A
Plasmid: PFLAG-HDAC1	This paper	N/A
Plasmid: pFLAG-HDAC1-Δ1 (aa.1-109)	This paper	N/A
Plasmid: pFLAG-HDAC1-Δ2 (aa.109-160)	This paper	N/A
Plasmid: pFLAG-HDAC1-Δ3 (aa.1-33)	This paper	N/A
Plasmid: pFLAG-HDAC1-Δ4 (aa.33-109)	This paper	N/A
Plasmid: pFLAG-HDAC1-K8R	This paper	N/A
Plasmid: pFLAG-HDAC1-K10R	This paper	N/A
Plasmid: pFLAG-HDAC1-K31R	This paper	N/A
Plasmid: pFLAG-HDAC1-K50R	This paper	N/A
Plasmid: pFLAG-HDAC1-K58R	This paper	N/A
Plasmid: pFLAG-HDAC1-K66R	This paper	N/A
Plasmid: pFLAG-HDAC1-K74R	This paper	N/A
Plasmid: pFLAG-HDAC1-K89R	This paper	N/A
Plasmid: pFLAG-HDAC2	This paper	N/A
Plasmid: pFLAG-HDAC2-K75R	This paper	N/A
Plasmid: HA-UB	Dr. Bo Zhong	N/A
Plasmid: HA-UB-K48	Dr. Bo Zhong	N/A
Plasmid: HA-UB-K63	Dr. Bo Zhong	N/A
Plasmid: pLKO.1-puro	Sigma-Aldrich	SHC001

**Software and algorithms**		

Fiji (ImageJ)	National Institutes of Health	https://imagej.nih.gov/ij/
Zeiss Zen Blue 3.1	ZEISS	https://www.zeiss.com.cn/
Flowjo	FLOWJO	https://www.flowjo.com
Prism 8	GraphPad	https://www.graphpad.com
fastp	fastp	https://github.com/OpenGene/fastp
DESeq2	DESeq2	http://www.bioconductor.org/packages/release/bioc/html/DESeq2.html

**Other**		

Opti-MEM	Gibco	Cat# 31985062
DMEM, High Glucose	Gibco	Cat# 11965-092
RPMI 1640	Gibco	Cat# 22400071
Fetal Bovine Serum (FBS)	Gibco	Cat# 10437-028
PhosSTOP™ phosphatase inhibitor tablets	Roche	Cat# 4906837001
SYBR Premix Ex Taq	TaKaRa	Cat# RR420L
TRIzol	TaKaRa	Cat# AL31604A
PrimeScript RT reagent kit	TaKaRa	Cat# RR047A

### Resource availability

#### Lead contact

Further information and requests for resources and reagents should be directed to and will be fulfilled by the lead contact, Bei-Bei Chu, (chubeibei@henau.edu.cn).

#### Materials availability

New mouse lines generated in this study will be available upon request. All other reagents are available commercially. Sequence and product details are provided in the key resources table.

#### Data and code availability

Cell RNA-seq and LC-MS analysis data have been deposited at Mendeley and are publicly available as of the date of publication. Mendeley Data :https://doi.org/10.17632/hm86ys5vmk.1 are listed in the key resources table. Original western blot images have been deposited at Mendeley and are publicly available as of the date of publication. Mendeley Data :https://doi.org/10.17632/hm86ys5vmk.1 are listed in the key resources table.

All original data has been deposited at Mendeley and is publicly available as of the date of publication. Mendeley Data :https://doi.org/10.17632/hm86ys5vmk.1 are listed in the key resources table.

All original code additional information required to reanalyze the data reported in this paper is available from the lead contact upon request.

### Experimental model and study participant details

#### Ethics statement

Experiments involving animals were approved by the Committee on the Ethics of Animal Care and Use of Henan Agricultural University (HNND2022030812). The study was conducted in accordance with the Guide for the Care and Use of Animals in Research of the People’s Republic of China.

#### Mice

The *p53*^+/+^ and *p53*^˗/˗^ (B6.129S2-*Trp53*^*tm1Tyj/J*^) mice on a C57BL/6J background were purchased from the Jackson Laboratory. Genotyping was performed by PCR according to the manufacturer’s protocol. Female 6- to 8-week-old C57BL/6J mice were used for this study. Mice were housed in specific-pathogen-free facilities with a 12 h light-dark cycle and a controlled temperature of 22°C. Animal protocols were performed in accordance with the guide for the care and use of laboratory animals and the related ethical regulations at Henan Agricultural University.

#### Cells

THP-1, RAW264.7, 3D4/21, L929, and HEK293T cells were maintained in DMEM or RPMI 1640 supplemented with 10% FBS, 1% penicillin/streptomycin and 1% glutamine, at 37°C with 5% CO_2_. Transfection of DNA constructs was performed with Lipofectamine 3000 (Invitrogen) for HEK293T cells. The siRNA (Table S1) transfection was performed with Lipofectamine RNAiMAX (Invitrogen) for THP-1 and RAW264.7 cells.

THP-1 cells were differentiated overnight with RPMI 1640/10% FBS supplemented with 100 ng/mL phorbol-12-myristate 13-acetate (Sigma-Aldrich). After being washed three times with PBS, the cells were re-plated in RPMI 1640/10% FBS and allowed to recover for 2 days. For BMDM preparation, femurs and tibias were dissected from mice. Bone marrow was flushed from bones, and red blood cells were lysed with ACK lysis buffer. Debris was removed by passage of cells through a 70 μm cell strainer. Approximately 5.3 × 10^6^ bone marrow cells were cultured in 100-mm dishes in complete DMEM with 20% M-CSF-containing conditional medium from L929 cells for 6 days.

#### Viruses

The HSV-1 F strain was a gift from Chun-Fu Zheng (Department of Microbiology, Immunology and Infectious Diseases, University of Calgary, Calgary, Alberta, Canada).^59^ PRV-QXX was used as previously described.^60^ Viruses were propagated, and their titers were determined with plaque assays on Vero cells. HBAAV2-siControl and HBAAV2-siDHRS3 were prepared by HANBIO (Shanghai, China). For cell-based assays, cells were infected with HSV-1 (MOI = 0.1 or MOI = 1, as indicated) or PRV-QXX (MOI = 1). For *in vivo* studies, mice were intranasally infected with HSV-1 (1 × 10^6^ pfu per mouse, or 1 × 10^7^ pfu per mouse as indicated), or injected with HBAAV2 (1.3 × 10^11^ vg per mouse) *via* tail vein injection.

### Method details

#### Sample preparation for metabolome

A 20 μL volume of 2-chloro-l-phenylalanine (0.3 mg/mL) dissolved in methanol as an internal standard and 1 mL of methanol:water (4/1, vol/vol) were added to each sample after homogenization. Samples were transferred to a 4 mL glass vial, 200 μL of trichloromethane was added to each vial, and samples were dispersed with a pipette. An ultrasonic homogenizer was used to disrupt the cells for 3 min at 500 W. All mixtures in each sample were transferred to 1.5 mL tubes, then extracted by ultrasonication for 20 min in an ice water bath. The extract was centrifuged at 16, 000 × *g* for 10 min at 4°C. Subsequently, 1 mL of supernatant in a glass vial was evaporated at room temperature. Then 200 μL of 1/4 (vol/vol) methanol and water was added to each sample, vortexed for 30 s and placed at 4°C for 2 min. Samples were centrifuged at 16, 000 × *g* for 15 min at 4°C. The supernatants (150 μL) from each tube were collected with glass syringes, filtered through 0.22 μm microfilters and transferred to LC vials. The vials were stored at -80°C until LC-MS or HPLC analysis.

#### LC-MS analysis

The samples were analyzed with an ACQUITY UPLC I Class system (Waters) coupled with a VION IMS QTOF mass spectrometer (Waters) with both ESI positive and ESI negative ion modes. Reversed-phase separation was performed with an ACQUITY UPLC HSS T3 column (100 mm × 2.1 mm, 1.8 μm). For the positive-ion mode, the ion spray floating voltage was set at 3.5 kV, whereas for the negative-ion mode, the voltage was set at -3 kV. The MS data were acquired in MSE mode. The TOF mass range was 125–1,000 Da. During the entire period, the mass accuracy was calibrated after every ten samples. Furthermore, the QC sample was analyzed after every ten samples to evaluate the stability of the LC-MS.

#### HPLC analysis

Reverse-phase chromatography was performed on an Agilent 1290 Infinity II instrument (Agilent Technologies). The chromatographic separation was performed on a Phenomenex C18 column (50 × 2.1 mm, 1.8 μm) at a flow rate of 10 μL/min. Methanol and 10 mM ammonium acetate buffer solution (1/4, vol/vol, pH 4.0 adjusted with acetic acid) was used as the mobile phase. UV detection was performed at 340 nm, and the column temperature was 40°C.

#### RNA-seq

RNA was extracted from THP-1 cells treated by vehicle or RA (10 μM) at 24 h post-treatment with TRIzol reagent (TaKaRa) according to the manufacturer’s protocol, with n = 3 biologically independent repeats per group. RNA-seq libraries were constructed with a TruSeq RNA Library Prep Kit (Illumina) according to the manufacturer’s instructions. The libraries were sequenced on the llumina Novaseq 6000 platform, and 150 bp paired-end reads were generated. Raw reads of fastq format were first processed with fastp, and low quality reads were removed to obtain clean reads. Differential expression analysis was performed with DESeq2.

#### Mouse disease scoring

Mice were scored for disease at the indicated times post infection. The scoring was performed in a blinded manner: symptoms associated with neurological disease (0: normal, 1: jumpy, 2: uncoordinated, 3: hunched/lethargic, 4: unresponsive/no movement); cerebellar ataxia score (0: walking along the ledge without losing balance, 1: occasionally losing footing but otherwise coordinated, 2: unable to use hind legs or landing on head rather than paws during descent, 3: unable to move on the ledge).

#### Immunoblotting analysis

Cells were harvested and lysed with lysis buffer (50 mM Tris–HCl, pH 8.0, 150 mM NaCl, 1% Triton X-100, 1% sodium deoxycholate, 0.1% SDS and 2 mM MgCl_2_) supplemented with protease and phosphatase inhibitor cocktail. Protein samples were separated with SDS-PAGE and then transferred to a membrane, which was incubated in 5% nonfat milk for 1 h at room temperature. The membrane was then incubated with primary antibodies overnight at 4°C, followed by horseradish-peroxidase-conjugated secondary antibodies for 1 h at room temperature. Immunoblotting results were visualized with luminata crescendo western HRP substrate (Millipore) on a GE AI600 imaging system.

#### RNA extraction and qRT-PCR

Total RNA was isolated with TRIzol reagent (TaKaRa) and subjected to cDNA synthesis with a PrimeScript RT reagent kit (TaKaRa). qRT-PCR was performed in triplicate with SYBR Premix Ex Taq (TaKaRa), according to the manufacturer’s instructions, and data were normalized to the level of *β-actin* expression in each individual sample. Melting curve analysis indicated the formation of a single product in all cases. The 2^˗ΔΔCt^ method was used to calculate relative expression changes. Primers used for qRT-PCR are listed in Table S1.

#### Comet assays

Cells were seeded in 6-well plates and treated as described. Normal melting point agarose (0.5%) was coated on frosted microscope slides. Approximately 10,000 cells in 10 μL DMEM were mixed with 75 μL low melting point agarose (0.7%), and the mixture was dripped onto the precoated normal melting point agarose layers. The third layers were prepared with 75 μL of 0.7% low melting point agarose. The cells were lysed in lysis buffer (10 mM Tris-HCl, pH 10.0, 2.5 M NaCl, 100 mM Na_2_EDTA, 1% Triton X-100 and 10% DMSO) for 2 h at 4°C. After lysis, the slides were placed in electrophoresis solution (300 mM NaOH, 1 mM Na_2_EDTA, pH >13) for 40 min, subjected to electrophoresis at 20 V (∼300 mA) for 25 min and subsequently neutralized with 0.4 mM Tris-HCl, pH 7.5. Finally, the cells were stained with PI (5 μg/mL) and evaluated on a Zeiss LSM 800 confocal microscope. DNA damage was measured in terms of tail moment in cometscore software.

#### Immunofluorescence analysis

Cells cultured on coverslips in 12-well plates were fixed with 4% (w/v) paraformaldehyde at room temperature for 20 min. After being washed with PBS three times, cells were permeabilized with 0.2% Triton X-100 for 20 min and then blocked with 10% FBS. The specific primary antibodies diluted in 10% FBS were added to the cells and incubated for 1 h at room temperature. After being washed with PBS three times, cells were incubated with the proper secondary antibodies diluted in 10% FBS for 1 h at room temperature. The nuclei were stained with DAPI for 5 min at room temperature, mounted with Prolong Diamond (Invitrogen) and examined on a Zeiss LSM 800 confocal microscope.

#### HBAAV2-mediated *Dhrs3* silencing

HBAAV2-siControl and HBAAV2-siDHRS3 were prepared by HANBIO Technology Co., Ltd. (Shanghai, China). A 100 μL volume containing 1.3 × 10^11^ vg HBAAV2-siControl or HBAAV2-siDHRS3 viruses was injected through the tail vein with a 29 gauge insulin syringe. Behavioral analysis was conducted at 32 days post-injection. Mouse sera were collected for HPLC analysis, and tissues were collected for immunoblotting, qRT-PCR and plaque assays at 34 days post-injection.

#### Lipid quantification

TC, TG and FFA were measured with a cholesterol assay kit (Applygen), triglyceride assay kit (Applygen) and LabAssay nonessential fatty acid kit (Wako) according to the manufacturer’s instructions. The values were normalized to the total cellular protein content.

#### Cytokine analysis

The medium from HP-1 cells was tested for IFN-β (R&D Systems) and IL-1β (R&D Systems) with ELISA kits according to the manufacturer’s instructions. Mouse serum was tested for IFN-β (PBL Assay Science) and IL-1β (R&D Systems) with ELISA kits according to the manufacturer’s instructions.

#### Plaque assays

Vero cells were cultured to confluency in six-well plates and inoculated with serially diluted viruses (10^−1^–10^−7^ fold) for 1 h at 37°C. The excess viral inoculum was removed by washing with PBS. Next, 4 mL of DMEM/1% methylcellulose was added to each well, and the cells were further cultured for 4–5 days. The cells were fixed with 4% paraformaldehyde for 15 min and stained with 1% crystal violet for 30 min before the plaques were counted.

### Quantification and statistical analysis

All data were analyzed in GraphPad Prism 8 software and a two-tailed Student’s *t*-test. *P* < 0.05 was considered statistically significant. Data are shown as the mean ± standard deviation for three independent experiments. For mouse survival studies, Kaplan–Meier survival curves were generated and analyzed for statistical significance.

### Additional resources

(See key resources table).
