# Supplementary material for: Alphaherpesvirus manipulates retinoic acid metabolism for optimal replication
Source: iScience. 2024 Jun 4;27(7):110144. doi: 10.1016/j.isci.2024.110144 (PMC11233922; doi:10.1016/j.isci.2024.110144)
Supplement: Document S1. Figures S1–S6 and Table S1 [file mmc1.pdf]

iScience, Volume *27*

Supplemental information

Alphaherpesvirus manipulates retinoic acid metabolism for optimal replication

Shengli Ming, Shijun Zhang, Jiayou Xing, Guoyu Yang, Lei Zeng, Jiang Wang, and Beibei Chu

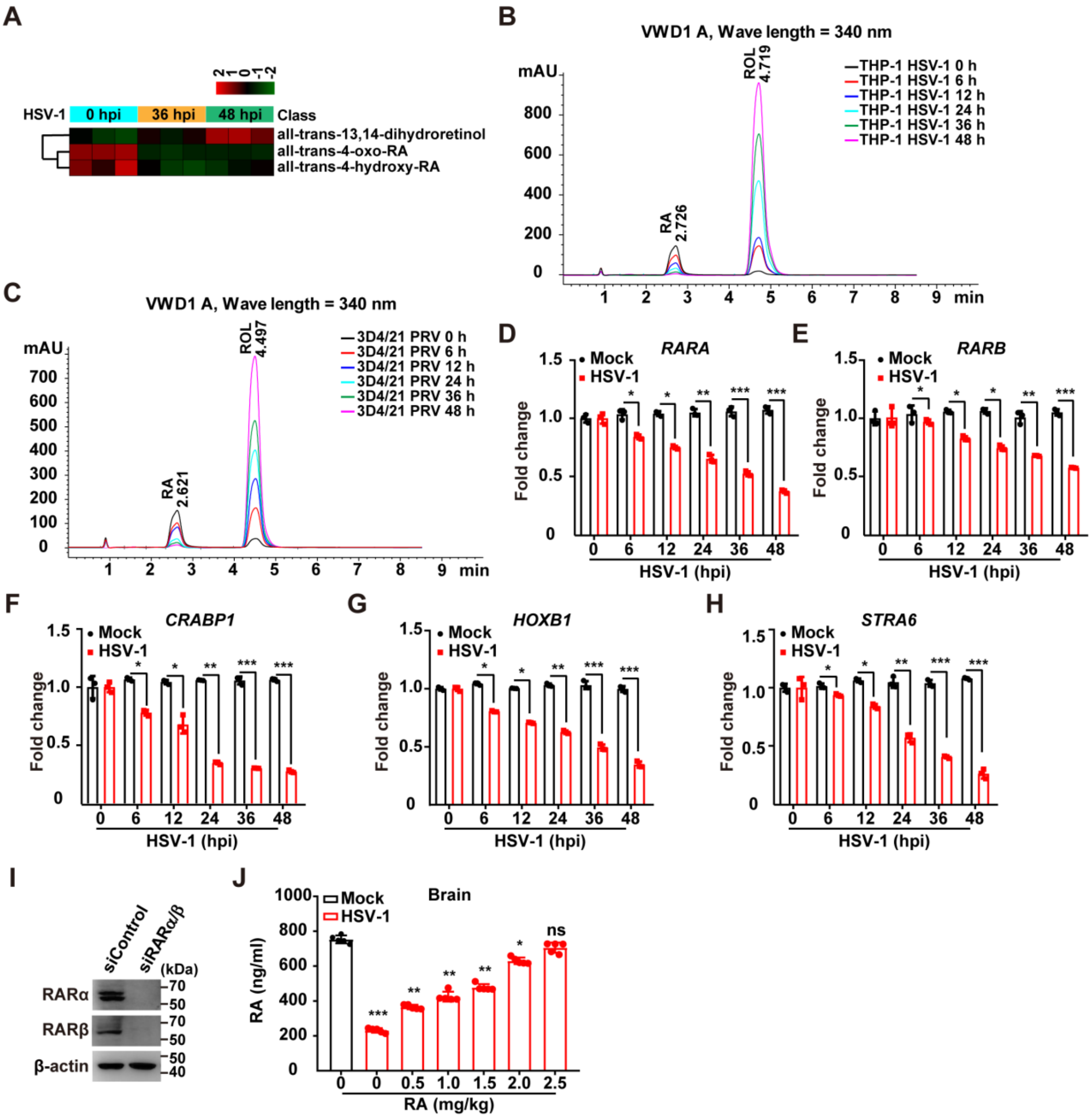

Fig. S1 (Related to Fig. 1 and Table S1). Viral infection inhibits RA synthesis.(A) Heat map of the fold change of indicated metabolites in THP-1 cells infected with HSV-1 (MOI = 0.1) for the indicated times.(B and C) Chromatogram of ROL and RA in THP-1 cells infected with HSV-1 (B, MOI = 0.1) or in 3D4/21 cells infected with PRV-QXX (C, MOI = 1) for the indicated times.(D-H) qRT-PCR analysis of *RARA* (D), *RARB* (E), *CRABP1* (F), *HOXB1* (G) and *STRA6* (H) in THP-1 cells mock-infected or infected with HSV-1(MOI = 0.1) for the indicated times.(I) Immunoblotting of the indicated proteins in siControl and siRARα/βTHP-1 cells.(J) RA in the brain in mice mock-infected or infected with HSV-1 (1×10^6 pfu per mouse) combined with treatment with RA (0–2.5 mg/kg) for 5 days.Data are expressed as mean ±SD of 3 independent experiments. p values were determined by Student's t test, *p < 0.05, **p < 0.01, ***p < 0.001. ns, no significance.

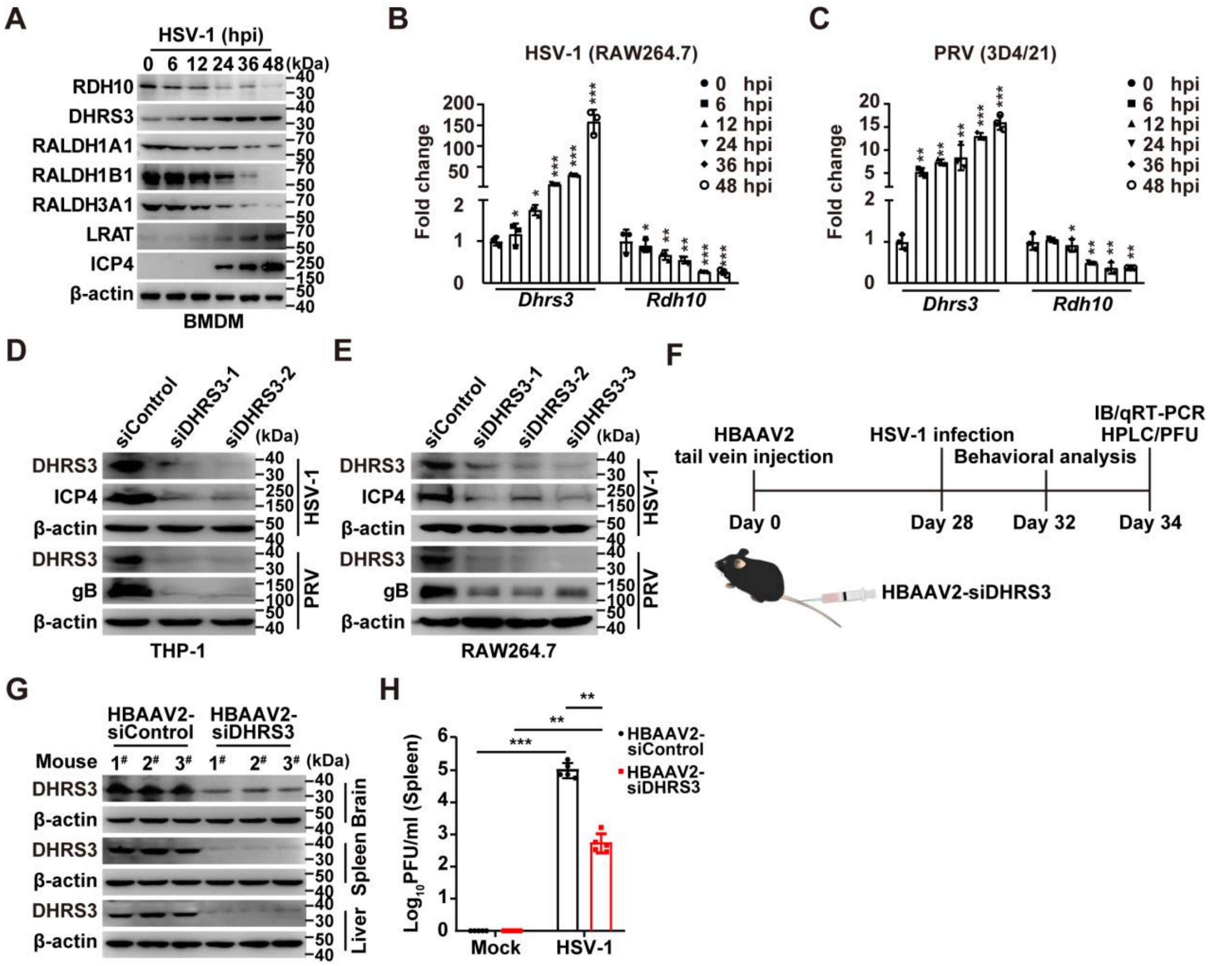

Fig. S2 (Related to Fig. 2 and Table S1). Knockdown of DHRS3 inhibits viral proliferation.(A) Immunoblotting of the indicated proteins in BMDM infected with HSV-1 (MOI = 0.1) for the indicated times.(B and C) qRT-PCR analysis of *Rdh10* and *Dhrs3* in RAW264.7 cells infected with HSV-1 (B, MOI = 1) or in 3D4/21 cells infected with PRV-QXX (C, MOI = 1) for the indicated times.(D and E) Immunoblotting of the indicated proteins in siControl and siDHRS3 THP-1 (D) or RAW264.7 (E) cells infected with HSV-1 (MOI = 0.1) or PRV-QXX (MOI = 1) at 24 hpi.(F) Schematic diagram of the protocol of HBAAV2-mediated DHRS3 knockdown and HSV-1 infection *in vivo*.(G) Immunoblotting of the indicated proteins in the brain, spleen and liver in mice infected with HBAAV2-siControl (1.3 × 10^{11} vg per mouse) or HBAAV2-siDHRS3 (1.3 × 10^{11} vg per mouse) for 28 days.(H) Viral titer analysis in the brain in mice from G.Data are expressed as mean ± SD of 3 independent experiments. *p* values were determined by Student's *t* test, **p* < 0.05, ***p* < 0.01, ****p* < 0.001.

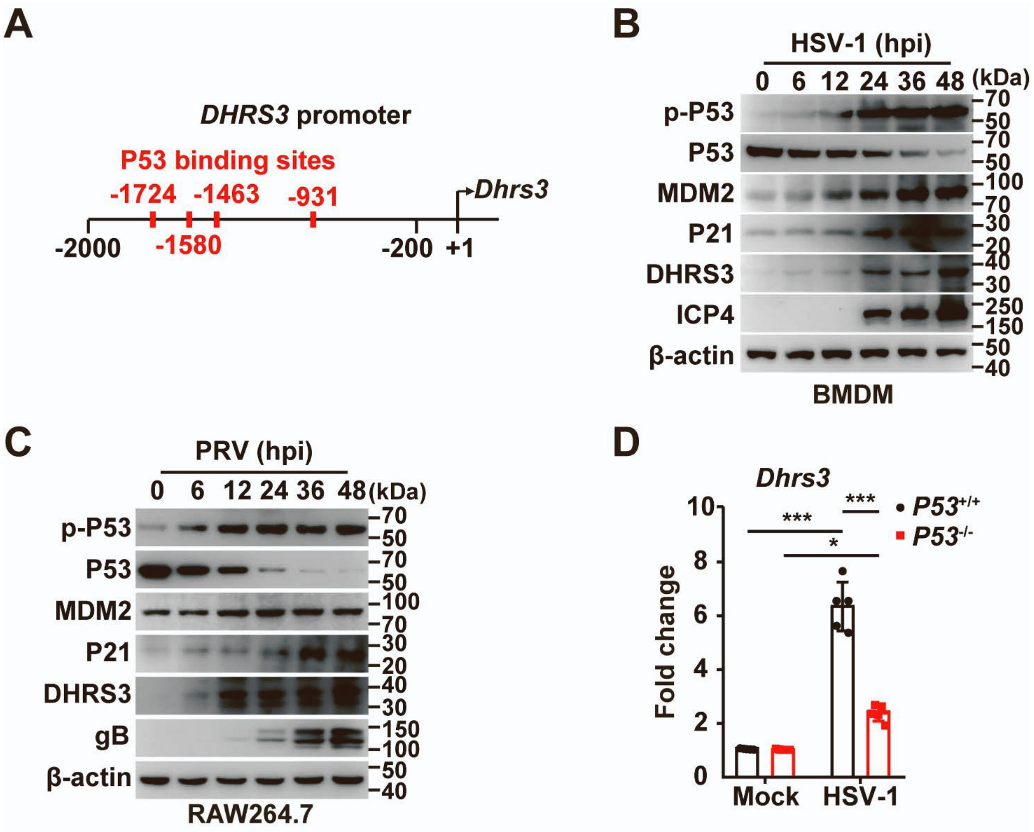

Fig. S3 (Related to Fig. 3 and Table S1). P53 is responsible for virus-induced upregulation of DHRS3.(A) Schematic diagram of the *DHRS3* promoter with potential P53 binding sites.(B) Immunoblotting of the indicated proteins in BMDM infected with HSV-1 (MOI = 0.1) for the indicated times.(C) Immunoblotting of the indicated proteins in RAW264.7 cells infected with PRV-QXX (MOI = 1) for the indicated times.(D) qRT-PCR analysis of *Dhrs3* in the brain of $P53^{+/+}$ and $P53^{-/-}$ mice mock-infected or infected with HSV-1 (1×10^6 pfu per mouse) at 5 days post-infection.Data are expressed as mean ± SD of 3 independent experiments. p values were determined by Student's t test, $^*p < 0.05$, $^{***}p < 0.001$.

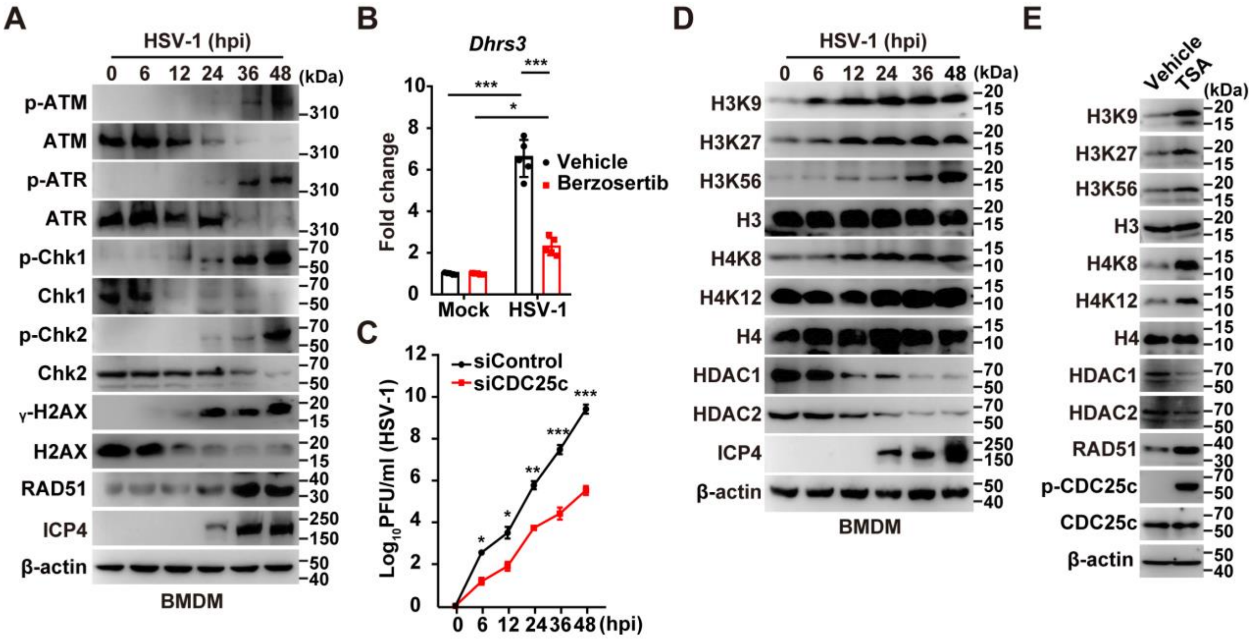

Fig. S4 (Related to Fig. 4 and Table S1). HSV-1 infection upregulates DHRS3 expression through DNA damage response.(A) Immunoblotting of the indicated proteins in BMDM infected with HSV-1 (MOI = 0.1) for the indicated times.(B) qRT-PCR analysis of *Dhrs3* in the brain in mice mock-infected or infected with HSV-1 (1×10^6 pfu per mouse) combined with treatment with vehicle or berzosertib (20 mg/kg) at 5 days post-infection.(C) Viral titer analysis siControl and siCDC25c THP-1 cells infected with HSV-1 (MOI = 0.1) for the indicated times.(D) Immunoblotting of the indicated proteins in BMDM from A. (E) Immunoblotting of the indicated proteins in THP-1 cells infected with HSV-1 (MOI = 0.1) combined with treatment with vehicle or TSA (1 μM) for the indicated times.Data are expressed as mean ± SD of 3 independent experiments. *p* values were determined by Student's *t* test, **p* < 0.05, ****p* < 0.001.

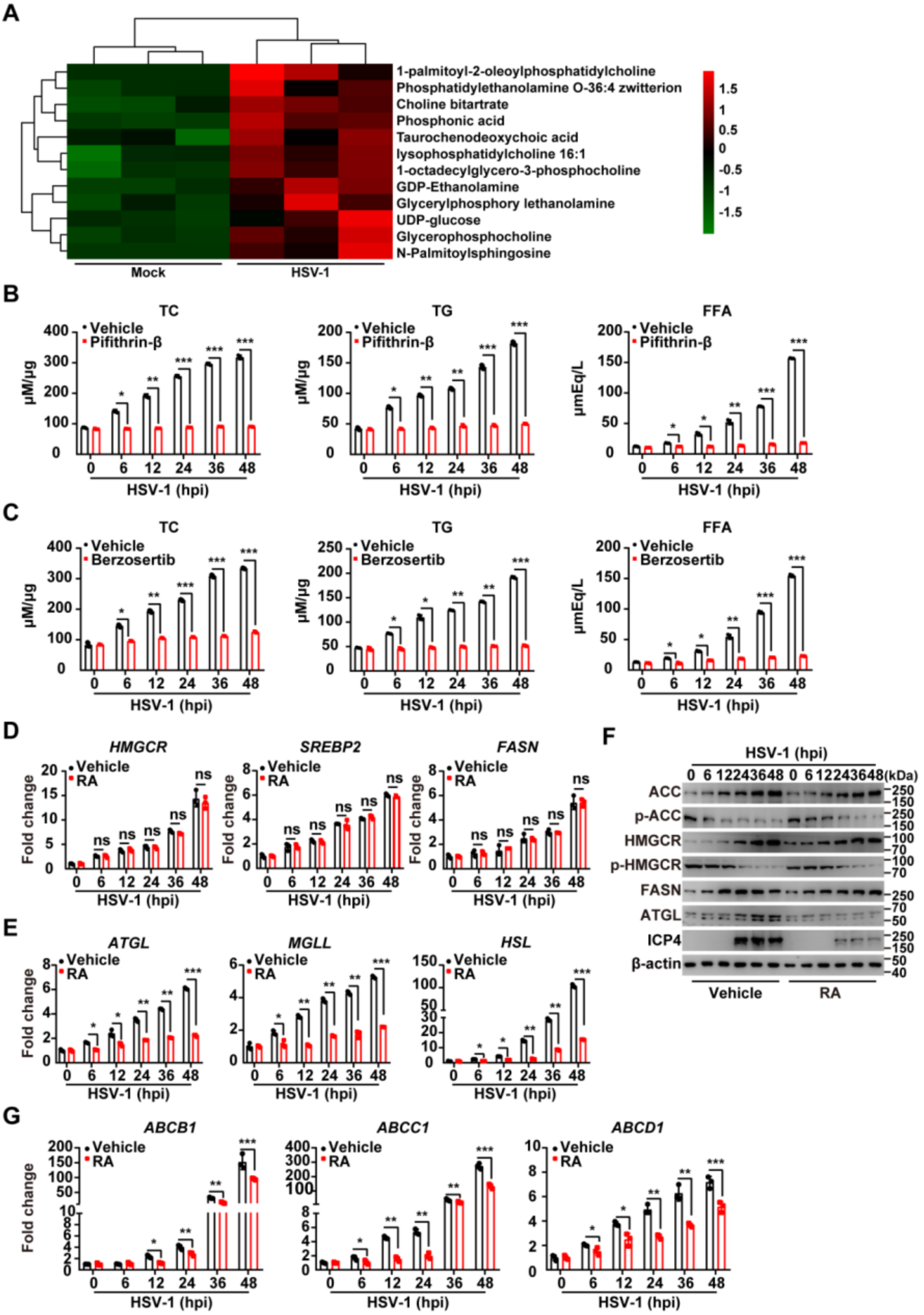

Fig. S5 (Related to Fig. 5 and Table S1). HSV-1 infection promotes lipid metabolism.(A) Heat map of the fold change of indicated metabolites in THP-1 cells infected with HSV-1 (MOI = 0.1) at 48 hpi.(B) TC, TG and FFA in THP-1 cells infected with HSV-1 (MOI =0.1) and treated with vehicle or pifithrin-β (10 μM) for the indicated times.(C) TC, TG and FFA in THP-1 cells infected with HSV-1 (MOI =0.1) and treated with vehicle or berzosertib (50 nM) for the indicated times.(D) qRT-PCR analysis of *HMGCR*, *SREBP2* and *FASN* in THP-1 cells infected with HSV-1 (MOI = 0.1) and treated with vehicle or RA (10 μM) for the indicated times.(E) qRT-PCR analysis of *ATGL*, *MGLL* and *HSL* in THP-1 cells infected with HSV-1 (MOI = 0.1) and treated with vehicle or RA (10 μM) for the indicated times.(F) Immunoblotting of the indicated proteins in THP-1 cells infected with HSV-1 (MOI = 0.1) and treated with vehicle or RA (10 μM) for the indicated times.(G) qRT-PCR analysis of *ABCB1*, *ABCC1* and *ABCD1* in THP-1 cells infected with HSV-1 (MOI = 0.1) and treated with vehicle or RA (10 μM) for 24 h.Data are expressed as mean ±SD of 3 independent experiments. p values were determined by Student's t test, $*p < 0.05$, $**p < 0.01$, $***p < 0.001$.

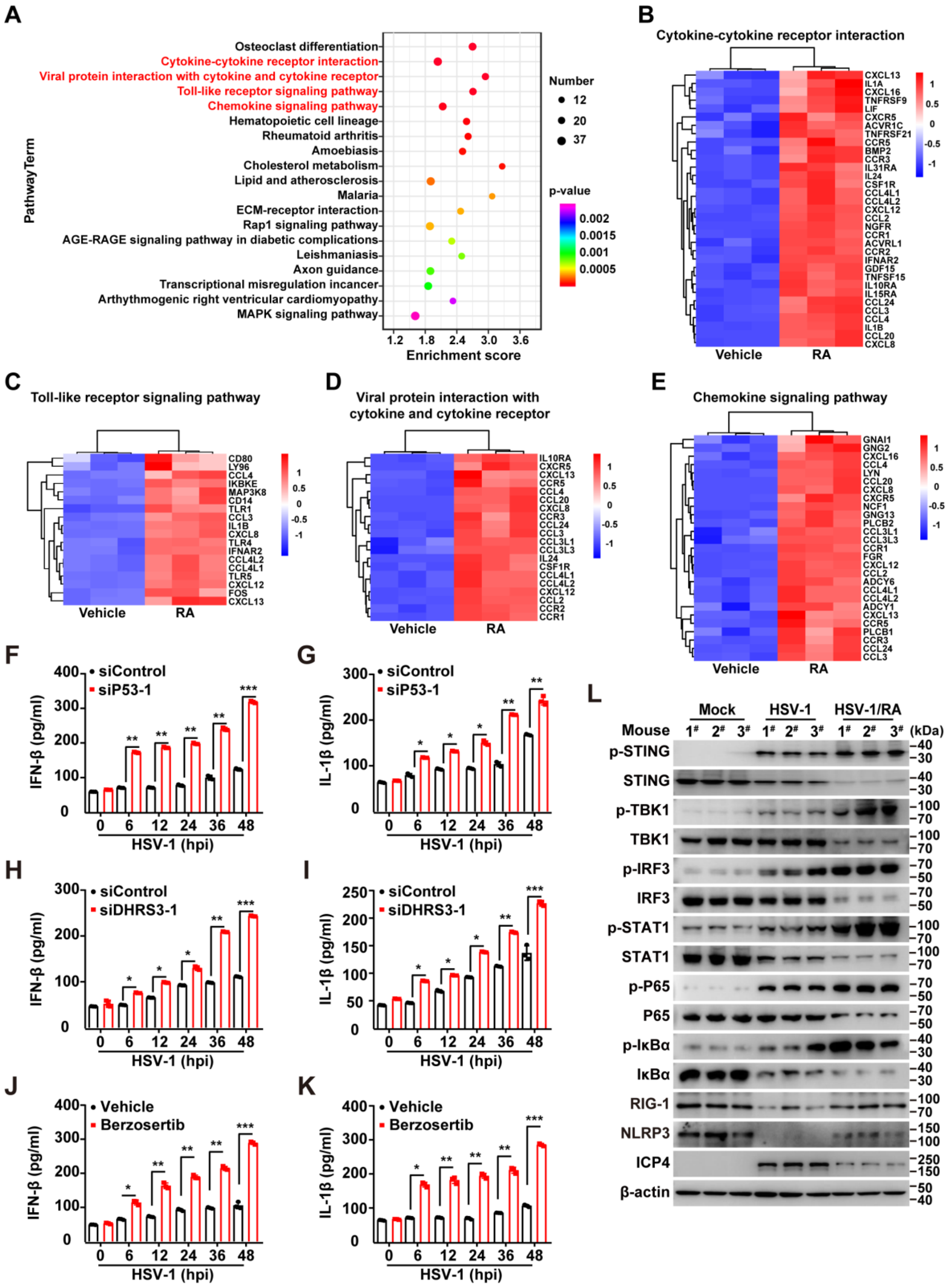

Fig. S6 (Related to Fig. 6). RA enhances the antiviral innate immunity.(A) KEGG-enriched pathways of THP-1 cells treated with vehicle or RA (10 μM) for 24 h.(B-E) Heat map of the fold change of indicated genes in THP-1 cells treated with vehicle or RA (10 μM) for 24 h.(F and G) IFN-β (F) and IL-1β (G) secretion from siControl and siP53 THP-1 cells infected with HSV-1 (MOI = 0.1) for the indicated times. (H and I) IFN-β (H) and IL-1β (I) secretion from siControl and siDHRS3 THP-1 cells infected with HSV-1 (MOI = 0.1) for the indicated times.(J and K) IFN-β (J) and IL-1β (K) secretion from THP-1 cells infected with HSV-1 (MOI = 0.1) and treated with vehicle or berzosertib (50 nM) for the indicated times.(L) Immunoblotting of the indicated proteins in the liver in mice mock-infected or infected with HSV-1 (1×10^6 pfu per mouse) combined with treatment with vehicle or RA (2.5 mg/kg) for 5 days.Data are expressed as mean ± SD of 3 independent experiments. p values were determined by Student's t test, *$p < 0.05$, **$p < 0.01$, ***$p < 0.001$.

Table S1. List of siRNAs and primers used in this study. Related to the STAR Methods.

Genes	Forward (5′-3′)	Reverse (5′-3′)
siControl	UUCUCCGAACGUGUCACGUTT	ACGUGACACGUUCGGAGAATT
Human siRARα	GCAGUUCUGAAGAGAUAGUTT	ACUAUCUCUUCAGAACUGCTT
Human siRARβ	GCCACCAAGUGCAUUAUUATT	UAAUAAUGCACUUGGUGGCTT
Mouse siDHRS3 -1	GCGCCAGAAAGAUUGUUCUTT	AGAACAAUCUUUCUGGCGCTT
Mouse siDHRS3-2	CCUGUAUGAACACCUUUAATT	UUAAAGGUGUUCAUACAGGTT
Mouse siDHRS3-3	CCAUGAAUAUCCUCAUUAUTT	AUAAUGAGGAUAUUCAUGGTT
Human siDHRS3-1	CCUAAUGGACAGUGAUGAUTT	AUCAUCACUGUCCAUUAGGTT
Human siDHRS3-2	GCCAUCGACUACUGCACAUTT	AUGUGCAGUAGUCGAUGGCTT
Human siP53-1	CCCGGACGAUAUUGAACAATT	UUGUUCAAUAUCGUCCGGGTT
Human siP53-2	GCAUGAACCGGAGGCCCAUTT	AUGGGCCUCCGGUUCAUGCTT
Human siP53-3	CCACUGGAUGGAGAAUAUUTT	AAUAUUCUCCAUCCAGUGGTT
Human siABCA1	CCUCCCUGUUUCUGAAGAATT	UUCUUCAGAAACAGGGAGGTT
Human siABCG1	GCAGAAGGGAAAUGGUCAATT	UUGACCAUUUCCCUUCUGCTT
Human Q-*RARα*	CTGAATCGAGCTGAGAGGGC	TGTGAGCTGGCACTTTTCCT
Human Q-*RARβ*	TCGGAAGGCTTTTTGCAAGC	TTTTTCCCAGCCCCGAATCA
Human Q-*CRABP1*	AGCTGGCCAACGATGAACTT	ATTGGTAGGGGAAAAGGGCG
Human Q-*HOXB1*	GCCTTCGAGCTTTGAGGAGT	AAGGCAGCTGGTGCTATTGT
Human Q-*STRA6*	GGGACAAGTTTCCGGGAGAG	TCATCGATGTACCAGCTGCC
Human Q-*β-actin*	GCACAGAGCCTCGCCTT	CCTTGCACATGCCGGAG
HSV-1 Q-*ICP0*	CCTGTCGCCTTACGTGAACA	CCATGTTTCCCGTCTGGTCC
Human Q-*ABCA1*	CCTTGCCAGCAAGACGAAAC	CCTTTGCCATCCATCCCACT
Human Q-*ABCB1*	CAGATAAAAGAGAGGTGCAAC	CCTGTGGCAAAGAGAGCGAA
Human Q-*ABCC1*	ATGAGGGCACAGTTAAGGCG	TCGGTCATGTCGGGAGAGAT
Human Q-*ABCD1*	TGCAAAGGAAGGGCTACTCG	ATTCTCTGCTTCTCGCCACC
Human Q-*ABCG1*	TGTCTGATGGCCGCTTTCTC	CTGGACACCACCTCATCCAC
Human Q-*HDAC1*	CATCGCTGTGAATTGGGCTG	CCCTCTGGTGATACTTTAGCAGT
Human Q-*HDAC2*	TGGCCTTTCTGAGCTGATTT	AGCCACTGAAACAAGACTTCA
